# Does Indigenous health research have impact? A systematic review of reviews

**DOI:** 10.1186/s12939-017-0548-4

**Published:** 2017-03-21

**Authors:** Irina Kinchin, Janya Mccalman, Roxanne Bainbridge, Komla Tsey, Felecia Watkin Lui

**Affiliations:** 1 Centre for Indigenous Health Equity Research, School of Health, Medical and Applied Sciences, Psychology and Public Health Department, CQUniversity Australia, Cairns Square, Corner Abbott and Shields Streets, Cairns, Qld 4870 Australia; 2 The Cairns Institute, James Cook University, Building D3, Smithfield, Qld 4870 Australia

**Keywords:** Indigenous health, Research impact, Closing the gap, Research benefit, Research value, Translation, Decision making, Systematic review, Overview

## Abstract

**Background:**

Aboriginal and Torres Strait Islander Australians (hereafter respectfully Indigenous Australians) claim that they have been over-researched without corresponding research benefit. This claim raises two questions. The first, which has been covered to some extent in the literature, is about what type(s) of research are likely to achieve benefits for Indigenous people. The second is how researchers report the impact of their research for Indigenous people. This systematic review of Indigenous health reviews addresses the second enquiry.

**Methods:**

Fourteen electronic databases were systematically searched for Indigenous health reviews which met eligibility criteria. Two reviewers assessed their characteristics and methodological rigour using an a priori protocol. Three research hypotheses were stated and tested: (1) reviews address Indigenous health priority needs; (2) reviews adopt best practice guidelines on research conduct and reporting in respect to methodological transparency and rigour, as well as acceptability and appropriateness of research implementation to Indigenous people; and (3) reviews explicitly report the incremental impacts of the included studies and translation of research. We argue that if review authors explicitly address each of these three hypotheses, then the impact of research for Indigenous peoples’ health would be explicated.

**Results:**

Seventy-six reviews were included; comprising 55 journal articles and 21 Australian Government commissioned evidence review reports. While reviews are gaining prominence and recognition in Indigenous health research and increasing in number, breadth and complexity, there is little reporting of the impact of health research for Indigenous people. This finding raises questions about the relevance of these reviews for Indigenous people, their impact on policy and practice and how reviews have been commissioned, reported and evaluated.

**Conclusions:**

The findings of our study serve two main purposes. First, we have identified knowledge and methodological gaps in documenting Indigenous health research impact that can be addressed by researchers and policy makers. Second, the findings provide the justification for developing a framework allowing researchers and funding bodies to structure future Indigenous health research to improve the reporting and assessment of impact over time.

**Electronic supplementary material:**

The online version of this article (doi:10.1186/s12939-017-0548-4) contains supplementary material, which is available to authorized users.

## Background

The nature, quality and value of research evidence in positively impacting and raising Indigenous health status can justifiably be questioned when significant gaps in health outcomes between Indigenous people and benchmark populations persist globally [1, 2]. Questioning the way researchers do business is also being challenged by Indigenous people who continue to voice concerns about being over-researched without corresponding improvements in their health [3]. Relatively unchanged long-term health disparities for Indigenous populations strongly suggest that despite the plethora of health research, corresponding research impacts or benefits for Indigenous people are, at best, modest. As researchers, we have a moral obligation to be responsive to need and ensure that we deliver high impact research evidence from which societal benefits can flow. Research end-users such as policy-makers and practitioners need to know how much confidence they can place in research evidence to make smarter informed healthcare decisions [4]. In tight contemporaneous fiscal environments, ensuring quality evidence also makes sense from a cost perspective. In this paper, we focus on the quality of evidence reported in systematic reviews of Australian Aboriginal and Torres Strait Islander (hereafter respectfully Indigenous Australians) health research as a necessary prerequisite to effecting societal impacts and benefits. A systematic review of reviews on Indigenous Australian health research was conducted to assess the quality of research evidence and the extent to which impact and benefits were reported.

Following Bainbridge et al. [3] and for the purpose of this review of reviews we consider “research impact” and “research benefit” as discrete terms that are interrelated and interdependent. Research impact is defined as any area of influence flowing from the research endeavour including those that flow from research processes; these can be both positive and negative. Research benefit flows from areas of impact. In Indigenous health research, benefit is broadly defined as any elements of research that are advantageous or good; such as strengthening capacities, opening opportunities, or improving health outcomes that progress the interests that are valued by Indigenous people [5]. Benefit can be intended or unintended, inside or outside the immediate research environment, direct or indirect, tangible or intangible, immediate, short-term or longer-term; but benefit must be positively oriented and represented as elements of value derived from research [3]. Herein we use the term “impact” as the first step for attaining benefits, but look at its positively oriented nature, as in research benefits.

Silburn et al. [6] noted that the likelihood of favourable research impact and thus benefits is related to the ethical implementation of Indigenous health research processes. Maximising research impact is more probable when: 1) Indigenous people are direct and active participants; 2) issues of relevance to Indigenous people are identified and prioritised; 3) Indigenous peoples’ knowledges and perspectives are incorporated in processes and findings; 4) reporting of findings are meaningful; and 5) all potential end-users are engaged from the outset. Yet, it is unclear whether research is implemented ethically or whether the abundance of research conducted, purportedly to improve Indigenous health, is justified and produces quality evidence that benefits Indigenous people in ways that are meaningful and valued by them [3].

### Aim and hypotheses

This systematic review of reviews extends the author team’s prior work to elucidate the reporting of Indigenous research impact [3]. It synthesises evidence from multiple reviews in a variety of settings to provide information concerning the extent to which impact is examined across different health areas, programs and interventions. Based on the domains of the Research for Impact tool [4] developed for Australia’s national Indigenous health research organisation, the Lowitja Institute, a set of three a priori hypotheses were developed to test the extent to which published reviews make transparent the impact of research studies on Indigenous health that are included in their reviews. The Tool provides a step-by-step research logic framework designed to increase the value of research in Indigenous Australian health. It is based on the assumption that the implementation of the six objectives in the framework will lead to demonstrable short, medium and longer term impact. The performance or impact of research projects identified in this review of reviews were evaluated against these six objectives: 1) promoting Indigenous leadership and participation; 2) ensuring rigorous and transparent research priority setting; 3) ensuring the study type and design are appropriate to the research question(s); 4) ensuring respectful and rigorous implementation; 5) facilitating knowledge translation into policy and practice; and 6) determining the impact of the research versus its costs. Consistent with these objectives, the hypotheses developed for this systematic review of reviews are:

1. Reviews address Indigenous Australian health priority needs;
2. Reviews adopt best practice guidelines on research conduct and reporting in respect of methodological transparency and rigour, as well as acceptability and appropriateness of research implementation to Indigenous Australian peoples; and
3. Reviews explicitly report the incremental impacts of their included studies and translation of research.

The overarching intent of the systematic review of reviews is to influence the current research reporting and accountability frameworks and expectations towards greater focus on improving the quality of evidence and reporting research impact. This study complements other review of reviews of Indigenous health [7, 8] by focussing explicitly on the impact of research. It also extends the search scope from peer-reviewed to policy initiated reviews (resource sheets), considers reviews’ output over time, and provides characteristics of included studies and institutional affiliations. The review therefore provides researchers, policy-makers, health practitioners and other decision-makers with directions towards greater focus on reporting and demonstrating research impact–the knowledge they need to implement evidence-informed health and wellbeing improvements.

## Methods

In recent years, health research has been assessed through reviews of the literature. Reviews help to make evidence more accessible by bringing research together in one place [9]. We worked from the premise that judgements about the quality of evidence could best be assessed through systematic review methods. We followed the established PRISMA (Preferred Reporting Items for Systematic reviews and Meta-Analyses) guidelines [10] for designing and preparing the manuscript. An a priori protocol defined the eligibility criteria, information sources and search strategy, outlined the data collection process, synthesis of results and quality appraisal strategies.

### Eligibility criteria

Studies included: (1) any type of review, for example literature review or meta-analysis or meta-synthesis, meta-ethnography or narrative synthesis, systematic search or bibliometric analysis; (2) focused on Indigenous Australians’ health and wellbeing; (3) published between 1995 and 2014 (inclusive); (4) in the English language; and (5) available as full-text manuscripts.

Indigenous health and wellbeing was defined broadly as “the social, emotional and cultural wellbeing of the whole community in which each individual is able to achieve their full potential as a human being, thereby bringing about the total wellbeing of their community, but not just the physical wellbeing of an individual” [11]. This broad definition meant that studies were included if they addressed a health-related process such as mentoring, health promotion, or a determinant of health such as education or housing initiatives, provided they met all other criteria. Reviews that were international in scope and crossed over Indigenous and tribal populations across various countries (*n* = 8, 11%) and reviews that considered Indigenous people as a part of a rural or a socially disadvantaged population (*n* = 3, 4%) were also included, if they reported results separately for Indigenous Australians.

As a quality indicator, this systematic review of reviews was limited to peer-reviewed literature. However, it was acknowledged that grey literature “may contain rich texts and their inclusion may avoid publication bias” [12]. With this caveat in mind, one pertinent sub-section of the peer-reviewed grey literature, known as Closing the Gap Clearinghouse, was included. Resource sheets and issues papers in the Clearinghouse contained evidence on narrowly defined topics commissioned by the Australian Institute of Health and Welfare and the Australian Institute of Family Studies (https://aifs.gov.au/). The aim of the Closing the Gap policy was to achieve equality in health status and life expectancy between Indigenous and non-Indigenous Australians. Resource sheets were subjected to peer review processes and considered to be of high relevance to Indigenous policies and programs.

### Information sources

An accredited librarian working at a leading Australian university designed and carried out a comprehensive search strategy of: (1) 14 electronic databases: Informit, Indigenous Australia, Indigenous Studies Bibliography: AIATSIS, APAIS-ATSIS, FAMILY-ATSIS, Informit Indigenous Collection, ATSIHealth, Campbell Library, EBM Reviews/Cochrane DSR/ACP Journal club/DARE, PsycINFO & PsychARTICLES, Medline, Ovid, CINAHL, Sociological Abstracts, Australian Indigenous HealthInfoNet; and (2) the Closing the Gap Clearinghouse website with a catalogued repository of evidence based resource sheets.

### Search

The following terms were searched in either title, abstract or MESH heading: systematic AND Australia AND review AND health AND (Aborigin* OR Indigenous OR “Torres Strait”). MESH terms comprised meta-analysis, oceanic ancestry group, Indigenous health services, Australia; subject terms included meta-analysis, Indigenous peoples OR Aborigines and health. The database search was performed in December 2014. The search was conducted separately for each database and use database specific search strings.

Discussions with experts established that systematic reviews in the field emerged as a common research type only in recent years. Therefore, selected period from 2004 to 2014 (inclusive) and from 1995 to 2014 for Medline were considered appropriate to provide comprehensive coverage of relevant reviews. The search of the Closing the Gap Clearinghouse website was conducted in May 2015.

### Data collection and analysis

Information was extracted from each included review for: first author, year, title, institutional affiliation and country of the first author; type of review; period searched (P), number of studies (K) and type of studies analysed; health area; target group: gender, age; reasons for the review; major findings (review authors’ conclusions); assessed research impact; review design; quality of included studies; compliance with Indigenous health research ethics.

Attention was devoted to understanding how researchers reported the impacts of their research for Indigenous people and how such impact was framed. The full texts of the reviews were searched for keywords such as impact, benefit, value, effect, consequence, assess* and translation.

Health areas were categorised using the socioeconomic determinants of health framework by Turrell et al. [13] and broadly divided into seven themes (areas): biological, psychosocial, health behaviour, healthcare system, health research, health inequality, setting and contexts. The authors of this study categorised reviews into as many health related areas as were relevant. Therefore, if a review fitted into more than one area, all were recorded.

An Excel (Microsoft Corporation) Datasheet 2013 was utilised to record the data items. One review author (IK) extracted the data from included studies and the second author (KT) cross-checked the extracted data. Two authors (IK, KT) independently assessed the included reviews. The coefficient of agreement was *k* = 85%, *z* = 16.15, *p* < 0.001, indicating a substantial level of agreement on extracted and recorded data items [14]. Discrepancies were resolved by discussion.

### Quality appraisal

Quality appraisal comprised a six-step analysis. Step 1: type of review was determined and considered as a quality measure [15]. Step 2: the authors ascertained the use of reporting guidelines, for example Preferred Reporting Items for Systematic reviews and Meta-Analyses (PRISMA) or any other relevant guidelines. Step 3: the reviews identified in the previous step were assessed with the AMSTAR tool [16], a validated measurement tool for assessing the methodological quality of reviews. The tool contains 11 questions (domains) (see Additional file 1). Each included review was assessed against all the domains with assessments labelled as ●, ◐ or ○. Reviews that fully addressed the domain were labelled ●; reviews that addressed the domain to some extent were assigned ◐; and reviews that either did not provide enough information for quality analysis, or the answer could not be ascertained, were assigned ○. Step 4: the authors investigated whether reviews assessed quality of the primary studies they had included. Step 5: the authors examined methodological rigour of the reviews through utilisation of quality appraisal tools informed by ethical standards and guidelines for conducting research with Indigenous people for the purpose of identifying the existence and utility of such tools. Step 6: lastly and importantly, the authors examined whether reviews documented Indigenous leadership and/or participation in research. Full texts of the reviews were examined for acknowledgment of Indigenous origin among authors and cross-checked by two authors (RB, JM) of this review.

### Synthesis of results

Due to the heterogeneity of health areas, interventions and outcomes, a quantitative summary measure of the results was not planned. Instead, descriptive analysis was used to synthesise results which were then presented according to the three hypotheses outlined in the introduction.

## Results

### Reviews included, and published over time

**Fig. 1:**
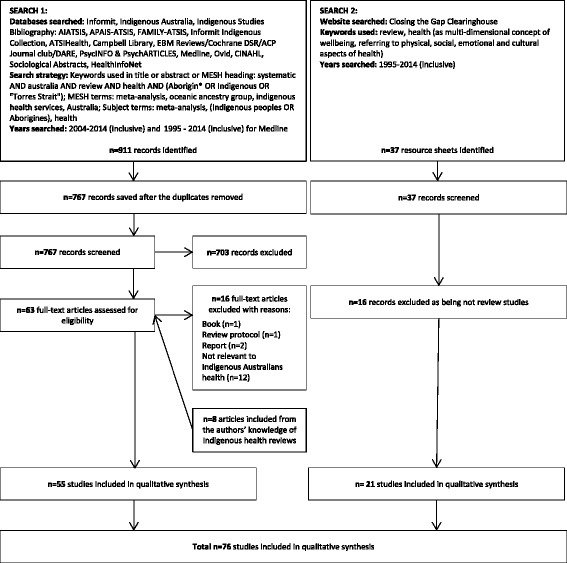
Search results

The initial search yielded 911 citations and 37 Closing the Gap Clearinghouse reports. Seven hundred sixty-seven remained after adjusting for duplicates. Of these, 703 citations and 16 Closing the Gap Clearinghouse reports were discarded based on the pre-specified inclusion and exclusion criteria. The full text of the remaining 84 citations (63 journal articles and 16 Closing the Gap Clearinghouse reports) were examined in more detail (Fig. 1).

Overall, 76 studies were included in the qualitative synthesis (see Additional file 2). Of these 76 reviews, 55 were journal articles and 21 were Closing the Gap Clearinghouse reports. The 76 reviews contained evidence from more than^1^ 5636 primary publications including randomised control trials, cohort studies, qualitative studies, and other designs. The average search period of the reviews was 17.5 years (SD^2^ 12, MD 17, Min 1, Max 62).

**Fig. 2:**
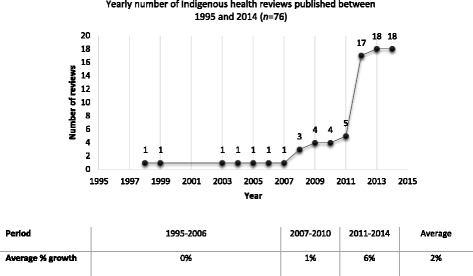
Trend in Indigenous health reviews published between 1995 and 2014

Over the 10-year period from 1995 to 2014, the number of Indigenous health reviews grew on average by 2%. Between 2011 and 2014, however, the average percentage growth was much higher, calculated to be 6% (Fig. 2).

### Characteristics of included primary studies

Descriptive studies dominated the list of primary publications, comprising a staggering 83% (*n* = 1,522). Intervention studies comprised the remaining 17% primary studies; there were no measurement studies found. Only two per cent of the primary studies (*n* = 31) were randomised control trials (RCTs) and five per cent (*n* = 100) were non-RCTs comparison studies of strategies or groups.

The demographics of the Indigenous population was considered in some reviews, with 22 reviews gender-age specific. Six reviews focused on women, and only one on men. Children and youth were a focus of thirteen reviews.

### Institutional affiliation

**Fig. 3:**
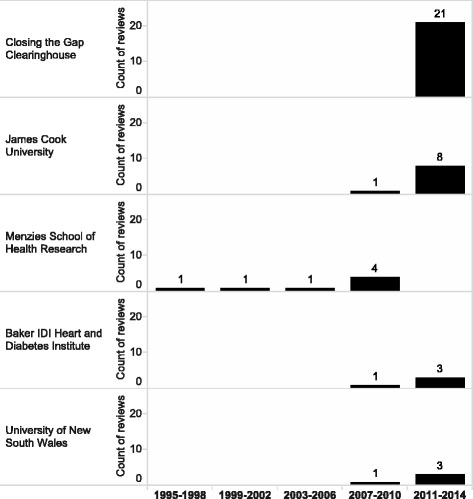
Top five institutions which published Indigenous health reviews between 1995 and 2014

Not surprisingly given the focus of this study and search terms, the largest proportion of the reviews came from Australia (70/76; 92%). Twenty-one Australian institutions contributed to the publication pool including [listed in descending order according to the number of published reviews in the field] the Closing the Gap Clearinghouse, James Cook University, Menzies School of Health Research, Baker IDI Heart and Diabetes Institute, University of New South Wales, Deakin University, Flinders University, University of Melbourne, University of Newcastle, University of Queensland, University of South Australia, University of Western Australia, Griffith University, Monash University, Queensland Health Mental Health Services, Southern Cross University, University of Sydney, University of Tasmania, University of Technology Sydney, Urbis Pty Ltd (Fig. 3).

**Fig. 4:**
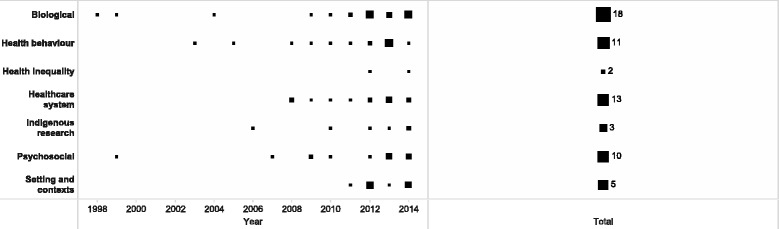
Distribution of reviews by health related area. Note. The size of the node is proportional to the number of reviews

Notably, Menzies School of Health Research was the first institution to start publishing reviews in the field [17]. James Cook University, Baker IDI Heart and Diabetes Institute and University of New South Wales only recently (after 2007) joined the pool of institutions that publish reviews on Indigenous health related topics. The Closing the Gap Clearinghouse contributed the largest number of reviews in the most recent period 2010–14 (Fig. 4). It is worth mentioning that most of the Closing the Gap Clearinghouse reports were produced and reviewed by researchers from national universities and other research institutions.

#### Hypothesis 1: reviews address Indigenous health priority needs

As mentioned in the methods section of this paper, the health priority areas were categorised according to the Turrell et al. [13] framework. Figure 4 displays the number of reviews segregated by the seven health related areas: biological, psychosocial, health behaviour, healthcare system, health research, health inequality, setting and contexts. The number attached to a node denotes the number of publications.

Of the 76 reviews, 22 (29%) related to biological health, 15 (20%) health behaviour, 13 (17%) health care system and 13 (17%) psychosocial health, 11 (14%) settings and contexts (Fig. 3). Biological and psychosocial health reviews were among the earliest published in the field. The “setting and context” reviews, covering topics on the social determinants of health such as education, parenting, housing, only gained research popularity since 2012 and had the highest average percentage growth thereafter at 9.1%. Only six reviews were related to Indigenous research output; including analysis of output and strategies for improving health and medical research. Only two reviews investigated health inequality. Table 1 outlines the foci of reviews within these categories.

**Table 1:** Indigenous health related areas of reviews

Health related area	Count^a^	Percent
Biological	20	26%
Respiratory system, incl. asthma (*n* = 3), ear disease (*n* = 1), otitis media (*n* = 1)	7	9%
Diabetes, incl. diabetes in pregnancy (*n* = 2) and association between cardiovascular disease and diabetes (*n* = 1)	4	5%
Obesity	2	3%
Cardio-metabolic disease	1	1%
Cardiovascular diseases	1	1%
Hepatitis B (HBsAg)	1	1%
Pregnancy and neonatal outcomes	1	1%
Sexually transmissible infections	1	1%
Strongyloides stercoralis infection	1	1%
Stomach cancer	1	1%
Health behaviour	15	20%
Tobacco smoking, incl. prevention	5	7%
Parasuicide self-harm and suicide, incl. prevention	4	5%
Physical activity and nutrition	3	4%
Alcohol misuse	1	1%
Health promotion tools	1	1%
Hygiene and hand washing	1	1%
Healthcare system, incl. services accessibility, delivery models, transfer and implementation of health services and programs	13	17%
Psychosocial	13	17%
Mental health	5	7%
Social and emotional wellbeing	4	5%
Other, incl. developmental outcomes in the early years of life, bush medicine treatment efficacy for cancer patients	4	5%
Setting and contexts	11	14%
Education	6	8%
Parenting, early childhood education	2	3%
Housing	2	3%
Employment with a disability	1	1%
Indigenous (Indigenous peoples’) research, incl. analysis of output, strategies for improving health and medical research	6	8%
Health inequality	2	3%

^a^Some reviews related to multiple health areas simultaneously

#### Hypothesis 2: reviews adopt best practice guidelines on research conduct and reporting in respect of methodological transparency and rigour, as well as acceptability and appropriateness of research implementation to Indigenous peoples

As mentioned in the methods, quality appraisal comprised a six-step analysis of: 1) types of reviews; 2) utilisation of reporting guidelines; 3) methodological quality of reviews; 4) quality of primary studies; and 5) methodological rigour from Indigenous perspective; 6) Indigenous leadership and participation in research.

##### Step 1. Types of reviews

Our categorisation of review methods, using the verbatim description provided in each review, found that the most frequently adopted approach was systematic synthesis of literature reviews (36/76; 47%). A further 27/76 reviews (36%) were termed “systematic reviews” (Table 2). These claim to have a rigorous methodology for searching, evaluating and reporting standards. The quality of these “systematic reviews” was assessed in step 3 of the analysis. Only four/76 reviews (5%) applied a meta-analysis, and only five/76 (7%) used a qualitative synthesis such as meta-synthesis of the evidence, meta-ethnography, narrative synthesis or an integrative review.

**Table 2:** Verbatim description of types of reviews used to synthesise Indigenous health reviews

Type of review	Count; %	Reference
Systematic approaches		
Systematic review	27; 36%	[17, 18, 20, 21, 26, 33–39, 42, 55–68]
Systematic review of the quantitative literature, descriptive studies, epidemiology and risk factors or intervention review	4; 5%	[43, 69–71]
Systematic search	2; 3%	[23, 27]
Critical review^a^	2; 3%	[2, 72]
Rapid review^a^	1;1%	[73]
Synthesised approaches		
Meta-analysis	4; 5%	[19, 24, 25]
Meta-synthesis of the evidence, meta-ethnography or narrative synthesis	3; 4%	[22, 28, 41]
Integrative review	2; 3%	[74, 75]
Others		
Resource sheet	15; 20%	[49, 50, 76–88]
Literature review (or review, review of the evidence, review of the literature)	13; 17%	[40, 44–46, 48, 51, 89–95]
Contextual review	1; 1%	[47]
Bibliometric analysis	1; 1%	[15]
Brief review	1; 1%	[96]

^a^With retained principles of a systematic review, including identified search engines, specified inclusion and exclusion criteria, and the quality assessment of retained literature

Other forms of reviews included resource sheets, a special type of inquiry published by the Closing the Gap Clearinghouse; literature review (or a review, review of the evidence, review of the literature); contextual review; bibliometric analysis; or brief review. By definition, these forms of reviews did not follow a systematic approach, including identified search engines, specified inclusion and exclusion criteria, and the quality assessment of retained literature.

##### Step 2. Utilisation of reporting guidelines

Our assessment of the reporting guidelines found that 52/76 reviews (68%) did not report on following any guidelines, and only 20/76 reviews (26%) specified and utilised a guideline such as, for example, PRISMA. Furthermore, 17/27 systematic reviews (63%) did not report on following review guidelines. Among 20 reviews that adhered to the reporting guidelines, 40% related to biological or clinical health; leaving the research on social determinants of health largely of poor methodological quality.

##### Step 3. Methodological quality of reviews

Our ascertainment of those 20 reviews, which reported following review guidelines, found that 15 (75%) scored “●–fully addressed” on more than six domains of the AMSTAR tool (Table 3). Nineteen of the 20 reviews (95%) were published in the last 5 years suggesting that review authors were only recently started adopting methodological rigour.

**Table 3:** Methodological quality assessment using AMSTAR measurement tool

First author	1.‘A priori’ design	2. Study selection and extraction	3. Literature search	4. Grey literature	5. List of studies	6. Study characteristics	7. Quality assessment	8. Methodological rigour	9. Pooled results	10. Publication bias	11. Conflict of interest
McDonald E, Bailie R, Brewster D and Morris P [33]	○	●	●	●	◐	●	●	●	●	○	●
Arnold M, Moore SP, Hassler S, Ellison-Loschmann L, Forman D and Bray F [55]	○	○	●	●	◐	●	○	●	○	○	●
Azzopardi PS, Kennedy EC, Patton GC, Power R, Roseby RD, Sawyer SM and Brown AD [56]	○	○	●	●	◐	●	●	●	○	○	●
McCalman J, Tsey K, Wenitong M, Wilson A, McEwan A, James YC and Whiteside M [28]	○	○	●	●	◐	●	○	○	●	◐	●
Gould GS, Munn J, Watters T, McEwen A and Clough AR [22]	○	●	◐	○	◐	●	●	●	●	◐	●
Shah PS, Zao J, Al-Wassia H and Shah V [24]	○	◐	●	●	◐	●	●	●	●	◐	●
Chang AB, Taylor B, Masters IB, Laifoo Y and Brown Alexander DH [43]	●	●	●	●	●	●	●	●	●	◐	●
McCalman J, Tsey K, Bainbridge R, Rowley K, Percival N, O’Donoghue L, Brands J, Whiteside M and Judd J [23]	●	◐	●	●	◐	●	●	●	○	◐	●
Clifford AC, Doran CM and Tsey K [26]	○	●	●	●	◐	●	●	●	NA	◐	●
McCalman J, Tsey K, Clifford A, Earles W, Shakeshaft A and Bainbridge R [27]	○	●	●	●	◐	●	●	●	NA	○	●
McCalman J, Bridge F, Whiteside M, Bainbridge R, Tsey K and Jongen C [61]	●	●	●	●	◐	●	◐	●	○	○	●
Calabria B, Clifford A, Shakeshaft AP and Doran CM [21]	○	●	●	○	◐	●	●	●	NA	◐	●
Ospina MB, Voaklander DC, Stickland MK, King M, Senthilselvan A and Rowe BH [25]	●	◐	●	●	◐	●	●	●	●	◐	●
Graham S, Guy RJ, Cowie B, Wand HC, Donovan B, Akre SP and Ward JS [19]	○	○	●	◐	◐	●	○	○	●	○	●
Adegbija OO and Wang ZQ [14]	○	●	●	○	◐	●	○	○	●	○	●
Lyons JG, O’Dea K and Walker KZ [20]	○	●	●	◐	◐	●	●	●	○	○	●
Laws R, Campbell KJ, van der Pligt P, Russell G, Ball K, Lynch J, Crawford D, Taylor R, Askew D and Denney-Wilson E [18]	●	●	●	◐	◐	●	●	●	NA	◐	●
Miller A, Smith ML, Judd J and Speare R [68]	○	○	●	●	◐	●	○	○	○	○	●
Banbury A, Roots A and Nancarrow S [73]	●	●	●	●	◐	●	●	●	○	○	○
Bainbridge R, Tsey K, McCalman J and Towle S [35]	○	●	●	●	◐	●	●	●	○	○	●
●–equivalent to yes in the original tool specification the paper fully addressed the domain	6 (30%)	10 (50%)	19 (95%)	14 (70%)	1 (5%)	20 (100%)	14 (70%)	15 (75%)	8 (40%)	0	19 (95%)
○–the paper did not address the domain at all or can’t answer	14 (70%)	7 (35%)	0	3 (15%)	0	0	5 (25%)	4 (20%)	8 (40%)	11 (55%)	1 (5%)
◐–the paper addressed the domain to some extent	0	3 (15%)	1 (5%)	3 (15%)	19 (95%)	0	1 (5%)	1 (5%)	0	9 (45%)	0

Reviews most commonly adhered to the following criteria: 3. Literature search, 4. Grey literature, 6. Study characteristics, 7. Quality assessment, 8. Methodological rigour and 11. Conflict of interest. Most reviews (95%) performed a comprehensive literature search and provided sufficient information, such as search terms, dates searched and justified search restrictions, for reproduction of the study. As well, most stated whether they included grey literature or unpublished studies and reports. In some reviews, grey literature was not included but listed as a limitation [18, 19] or was excluded but not justified [20]. In only three reviews, it could not be ascertained whether authors included any grey literature at all [14, 21, 22]. Most studies (70%) appraised the scientific quality and successfully incorporated the results of the analysis in the conclusions to the review. All reviews (100%) reported the characteristics of the included studies in an aggregated form such as a table, or a graph. As well, 95% (*n* = 19) of reviews acknowledged sources of funding.

The most poorly addressed domains (less or equal to 50% of reviews fully addressed the domain), were: 1.‘A priori’ design, 2. Study selection and extraction, 5. List of studies, 9. Pooled results, and 10. Publication bias (Table 3). Only 30% of the reviews met the criterion requiring an a priori protocol that defines the search strategy, sets the study selection criteria, outlines quality assessment and data extraction procedures, and plans the analysis of the study results. Half of the reviews did not engage the required number (at least two) of independent data extractors, nor stipulated a consensus procedure for disagreements. Some reviews partly met the criterion by employing one data extractor [23–25]. A staggering 95% of the reviews did not provide a list of excluded studies, reporting only the total number instead. Most reviews (40%) failed to explain the method utilised to combine the findings of primary studies. In some cases, reviews did not provide the method, but acknowledged as a limitation that, for example, meta-analysis as a form of synthesis, was improper to apply due to the heterogeneity [18, 21, 26, 27]. The majority of reviews did not address the domain “Publication bias” or addressed it partially. For example, one mentioned, but did not assess, the potential for publication bias [28]. Other reviews addressed the selection bias, usually as a criterion in the assessment tool [18, 22, 23, 25, 26], or attrition bias [24] instead.

Results of the analysis show that while the majority of the reviews (95%, 72 of 76) have mentioned search criteria and provided sufficient information for reproduction of the study [reproducible research is a key to new discoveries [29]], important caveats remain including lack of a priori design, reporting on study selection and extraction, listing of studies and pooled results and assessing publication bias.

##### Step 4. Quality of primary studies

Our ascertainment of the primary studies found that the most prevalent quality assessment tools were the Quality Assessment Tool for Quantitative Studies [30], CASP Tool For Qualitative Studies [31] and National Health and Medical Research Council (NHMRC) levels of evidence scale [32]. Forty nine/76 reviews (64%) did not use the tools to assess the quality of their primary studies. For those that did (27/76; 36%), the assessment included overall quality appraisal by hierarchy of evidence, assessment of the risk of bias, or assessment by study design.

Overall, critical assessment revealed that the majority of studies were of moderate [20–22, 25] to poor quality [23, 33–35], with weak study designs [36] and lack of consistently strong methodology across the majority of applied criteria [26]. According to the assessment by the hierarchy of evidence, most studies were of level IV, the lowest level of evidence [37–40]; that is, from case series, either post-test or pre-test/[and] post-test; or level III-3 [41] obtained from historical cohort studies, two or more single arm studies or interrupted time series without a parallel control group. Assessments of the risk of bias varied, from low [42] to moderate [24] and high [41] risk. Lack of blinding in some outcomes and insufficient sample size for the primary outcome [43] were primary reasons for the high risk of bias.

##### Step 5. Methodological rigour from Indigenous perspective

This step was addressed by determining whether reviews used quality appraisal tools that were informed by ethical standards and guidelines for conducting research with Indigenous people. A very high 95% of reviews (72/76) did not assess the ethics of research from an Indigenous perspective. The four reviews that did so, considered an Indigenous perspective via two means: by developing and utilising a context scale tailored to a specific health area and designed to appraise Indigenous research [37, 38, 44] or applying a general tool, not specifically designed to appraise Indigenous research [45].

##### Step 6. Indigenous leadership and participation in research

Indigenous leadership and participation in research was gauged by authorship of reviews as a lead author or as a co-author. Full texts of the reviews were examined for acknowledgment of Indigeneity among authors and cross-checked by two authors of this review. Twenty reviews/76 (26%) acknowledged or documented that their authors were Indigenous; in three reviews Indigenous authors were lead authors. Hence, nearly three quarter of the reviews were considered without Indigenous leadership or without acknowledging their Indigenous leadership or participation.

#### Hypothesis 3: Reviews explicitly report the incremental impacts of their included studies and translation of research

None of the reviews assessed the impact of research nor explicitly referred to research impact. Having said that, Van Schaik et al. [46] and Dudgeon et al. [44] utilised approaches that could potentially be used to capture research impact. Van Schaik et al. [46] incorporated Indigenous peoples’ specific knowledge by critically reviewing the literature associated with Indigenous peoples’ beliefs about cancer treatment. Dudgeon et al. [44] applied a three step assessment procedure to gauge the cultural appropriateness, the quality of the program evaluations, and whether the program achieved an effective outcome. Dudgeon et al. [44], Fromene et al. [47], Mildon et al. [48], Ware et al. [49], Ware et al. [50] and Nelson et al. [51] did not explicitly refer to the research impact but emphasised its importance and drew on the local cultural context and knowledge in conducting research, with a particular focus on the relevance of findings and recommendations to Indigenous people.

The authors of this systematic review of reviews noted that a designated journal section, for example “what are the new findings”, “lessons learned” or “what this paper adds”, usually prompted researchers to think explicitly about the incremental impact of their research and research translation. Table 4 provides some examples.

**Table 4:** Examples of journals prompting researchers to think along the lines of research impact and translation

Banbury et al. (2014) [73] *What this study adds:* ▸ E-health has the ability to increase access to services in rural and remote areas, substantially reduce travel costs and inconvenience to patients, and support professional development of health professionals. ▸ E-health should be implemented alongside change management processes. Shah et al. (2011) [24] *Implications for Policy* Several initiatives have been developed for policy implication based on findings of health outcomes disparity. These include “environmental” scan, identification of priority areas, ensuring management of jurisdictional overlap, training of local health care workers within community (community based partnership), identifying and promoting the “best” or “better” practices within the local context, and building and promoting effective partnership including development or expansion of existing midwifery educational programs for Indigenous people…	

## Discussion

This systematic review of reviews on Indigenous Australian health research analyses the quality of evidence and reporting of research impact in existing health reviews. We argue that if review authors explicitly address each of our three hypotheses, then the impact of research for Indigenous health would be explicated. While reviews are gaining prominence and recognition in Indigenous health research as an important methodological approach to dealing with ever growing amounts of research output, and are increasing in number, breadth and complexity, we found that currently, there is little reporting of the impact of health research for Indigenous people and that few met the criteria expected in the reporting of quality evidence. These findings raise questions about the relevance of these reviews for Indigenous people, their impact on policy and practice and how reviews have been commissioned, reported and evaluated.

Although the priorities for Indigenous health are arguable and vary from region to region, on the whole the findings of this systematic review of reviews partially support the first hypothesis: that reviews of Indigenous health address Indigenous health priority needs. This systematic review of reviews considers health broadly as a whole-of-life view associated with wellbeing of individuals and their communities and, to our knowledge, is among the first reviews that focuses not only on health from the Western perspective, but also on the important social determinants of health. To date, there is still a continuing predominance of reviews focussed on biological or clinical models of health which is promising but not sufficient for addressing Indigenous health. Social and emotional well-being encompasses all aspects of the social and emotional context of the person and their family, the historical and economic factors, including racism, oppression, trauma, grief, loss in its many forms, and the sequelae of the Stolen Generations, and it therefore allows for a more holistic framing of health that needs to be recognised in Indigenous research and its impact [47]. Further reviews of aspects of Indigenous mental health and social and emotional wellbeing are needed. Likewise cultural knowledge and context cannot be fully understood without Indigenous peoples’ involvement in research, participation and leadership which clearly has not been acknowledged and documented sufficiently in the analysed reviews.

Our second hypothesis, that reviews should adopt best practice guidelines on research conduct and reporting in respect of methodological transparency and rigour, as well as acceptability and appropriateness of research implementation to Indigenous peoples, was also rejected. Reviews have progressively become a mainstream methodology in Indigenous health research. Nevertheless, the current analysis reveals a clear misinterpretation as to what is understood by the term “review” and, in particular, “systematic review”. Nearly two thirds (63%) of the systematic reviews, which by definition claim to have a rigorous approach to searching, evaluating and reporting, have not reported nor followed any guidelines. Undertaking a systematic review requires a robust methodology. Authors who endeavour to undertake a systematic review are expected to meet detailed and rigorous requirements and follow guidelines or standards on the conduct and reporting. These include the development of an a priori protocol, which is submitted for peer review prior to the commencement of the review; undertaking rigorous searches which align with the inclusion and exclusion criteria specified in the protocol; and strict alignment with guidelines for the preparation of manuscripts for publication [52].

What was reported and how it was reported also varies considerably. The majority of the reviews were based on qualitative analyses of descriptive and intervention studies. Cooper et al. [53] observed that the traditional review process typically “lacks analytical precision because of biases associated with a reviewer’s idiosyncratic perspective, failure to assess the size of the effects reported by studies reviewed, and imprecise combination of the volume of evidence available across the studies reviewed”. According to Cooper et al. [53], a synthesised approach such as meta-analysis can offer a method that avoids some of the problems of the traditional literary summary. Synthesised approaches were underutilised in Indigenous health reviews. Having said that, reviews concerning Indigenous health are complex in nature with prevalent attribution issues; it is hard to establish the impact of a particular intervention if multiple simultaneous interventions are being delivered. Furthermore, Indigenous health is an area where randomised control trial interventions, with outcomes presented as quantified measures that can be subjected to meta-analysis, are not always ethically appropriate. All of these factors contributed to relatively weak evidence base in Indigenous health research.

The rigour of the research from the perspective of Indigenous people directly relates to scarcity of Indigenous leadership and/or participation. Concerns exist that the reporting of reviews has often been culturally inadequate. A few attempts have been made (four reviews out of 76) to assess the ethical appropriateness and rigour of the research from the perspective of Indigenous people, however, no systematic way of appraisal was found. Authors either developed and utilised a context scale tailored to a specific health area [37, 38, 44] or applied a generic tool, not specifically designed to align with an Indigenous research context [45]. This may reflect either, or both, a lack of awareness of reporting guidelines or tools informed by Indigenous peoples’ perspectives on ethically appropriate and rigorous research processes and outputs, or a perception that guidelines that first emerged from clinical medicine are not applicable in the social sciences where most Indigenous health research is being conducted. As pointed out [7], absence of such tools might lead to a waste of resources on reviews with limited relevance for Indigenous people. The marriage of health research to needs can only occur with consultation, engagement and the trust of Indigenous communities. We recognise the need for a cultural diversity tool specifically designed for conducting systematic reviews in the context of Indigenous health.

Finally, the third hypothesis, that reviews explicitly report the incremental impacts of included studies and research translation, was also rejected. Even though review authors might state an aim to influence policy or practice, often it was mostly unclear who or what they were attempting to influence, or the impact of their research. Dialogue at the nexus between research, policy, and practice is crucial if systematic reviews are to make a significant impact on practice. Instead, reviews need to be framed in such a way that they feed into the development and refinement of policy and practice guidelines which “interpret results to make knowledge of intervention effects available to a wide audience, including readers who are unfamiliar with the methodological and technical aspects of both primary research and research synthesis” [52, 54]. The identified lack of reporting research impact makes the decision by the Lowitja Institute to develop such a tool that would support planning and reporting impact of research over time responsive to the need and very timely [4].

### Limitations

While this paper provides a systematic review of the state of the Indigenous health research, because it is a systematic review of reviews, it does not include potentially useful primary studies that have not been included by the identified existing reviews. Reviews published prior to 1995 nor those published since 2015 have not been included. The search strategy could have been improved by adding a more sensitive search filter for Indigenous health related terms. It is possible that some reviews are excluded as a result; however, it is unlikely that the overall findings would be significantly different. We drew on the authors’ knowledge of Indigenous health research to identify additional studies. The authorship included one Aboriginal author (RB) and one Torres Strait Islander author (FWL). We acknowledge the limitations of our approach in addressing the impact of research though analysis of reviews rather than the original studies. We cannot rule out the possibility that original studies reported the impact of their research. However, if such had been reported in original studies, we assumed a high probability of reporting the impact by reviews as well. We encourage further research and analysis of original studies to address this limitation.

We also acknowledge the lack of a benchmark or a threshold figure for testing the research hypotheses. This was mainly due to the absence of any formal numeric criteria. Authors used their professional assessment to gauge whether hypotheses could be rejected or not. Lastly, the methodological quality of included reviews is assessed using the AMSTAR tool designed for studies in any population, not specifically Indigenous due to the nonexistence of Indigenous specific tool at the time of publication.

## Conclusion

The findings of our study serve two main purposes. First, we identified knowledge and methodological gaps in documenting Indigenous health research impact that can be addressed by researchers and policy makers. Second, the findings provide the justification for developing a framework allowing researchers and funding bodies to structure future Indigenous health research to improve the reporting of its impact; the framework that underpins principles of Indigenous leadership and participation, research capacity enhancement, methodological rigour of research and knowledge translation.

## Additional files

Additional file 1: AMSTAR measurement tool. (DOCX 14 kb)

Additional file 2: Summary of evidence from the Search 1. (DOCX 152 kb)

## Abbreviations

CASP tool: Critical Appraisal Skills Programme tool
Indigenous people: Aboriginal and Torres Strait Islander people
NHMRC: National Health and Medical Research Council
PRISMA guidelines: Preferred Reporting Items for Systematic reviews and Meta-Analyses guidelines
RCTs: Randomised control trials

## Footnotes

1 Not all reviews specified the number of included studies

2 SD–standard deviation; MD–median
