# Supplementary material for: Does Indigenous health research have impact? A systematic review of reviews
Source: Int J Equity Health. 2017 Mar 21;16:52. doi: 10.1186/s12939-017-0548-4 (PMC5361858; doi:10.1186/s12939-017-0548-4)
Supplement: Supplementary file 1 — AMSTAR measurement tool. (DOCX 14 kb) [file 12939_2017_548_MOESM1_ESM.docx]

**AMSTAR measurement tool**

Each included review was assessed against all the questions/domains of the tool with assessments labelled as ●, ◐ or ○. Reviews that fully addressed the domain were labelled ●; reviews that addressed the domain to some extent were assigned ◐;and reviews that either did not provide enough information for quality analysis, or the answer could not be ascertained, were assigned ○.

| **1. Was an 'a priori' design provided?**  The research question and inclusion criteria should be established before the conduct of the review |
| --- |
| **2. Was there duplicate study selection and data extraction?**  There should be at least two independent data extractors and a consensus procedure for disagreements should be in place |
| **3. Was a comprehensive literature search performed?**  At least two electronic sources should be searched. The report must include years and databases used (e.g. Central, EMBASE, and MEDLINE). Key words and/or MESH terms must be stated and where feasible the search strategy should be provided. All searches should be supplemented by consulting current contents, reviews, textbooks, specialized registers, or experts in the particular field of study, and by reviewing the references in the studies found |
| **4. Was the status of publication (i.e. grey literature) used as an inclusion criterion?**  The authors should state that they searched for reports regardless of their publication type. The authors should state whether or not they excluded any reports (from the systematic review), based on their publication status, language etc |
| **5. Was a list of studies (included and excluded) provided?**  A list of included and excluded studies should be provided |
| **6. Were the characteristics of the included studies provided?**  In an aggregated form such as a table, data from the original studies should be provided on the participants, interventions and outcomes. The ranges of characteristics in all the studies analysed e.g. age, race, sex, relevant socioeconomic data, disease status, duration, severity, or other diseases should be reported |
| **7. Was the scientific quality of the included studies assessed and documented?**  'A priori' methods of assessment should be provided (e.g., for effectiveness studies if the author(s) chose to include only randomized, double-blind, placebo controlled studies, or allocation concealment as inclusion criteria); for other types of studies alternative items will be relevant |
| **8. Was the scientific quality of the included studies used appropriately in formulating conclusions?**  The results of the methodological rigour and scientific quality should be considered in the analysis and the conclusions of the review, and explicitly stated in formulating recommendations |
| **9. Were the methods used to combine the findings of studies appropriate?**  For the pooled results, a test should be done to ensure the studies were combinable, to assess their homogeneity (i.e. Chi-squared test for homogeneity, I^2^). If heterogeneity exists a random effects model should be used and/or the clinical appropriateness of combining should be taken into consideration (i.e. is it sensible to combine?) |
| **10. Was the likelihood of publication bias assessed?**  An assessment of publication bias should include a combination of graphical aids (e.g., funnel plot, other available tests) and/or statistical tests (e.g., Egger regression test) |
| **11. Was the conflict of interest stated?**  Potential sources of support should be clearly acknowledged in both the systematic review and the included studies |
