# Supplementary material for: Does Indigenous health research have impact? A systematic review of reviews
Source: Int J Equity Health. 2017 Mar 21;16:52. doi: 10.1186/s12939-017-0548-4 (PMC5361858; doi:10.1186/s12939-017-0548-4)
Supplement: Supplementary file 2 — Summary of evidence from the Search 1. (DOCX 152 kb) [file 12939_2017_548_MOESM2_ESM.docx]

Summary of evidence from the Search 1

| # | First author, year, title, institutional affiliation, country | Type of review^[[1]](#footnote-1)^, period searched (P), number of studies (K) and type of studies analysed | Health area | Target group: gender, age | Reasons for the review | Major findings (review authors’ conclusions) | Research impact^[[2]](#footnote-2)^ | Review design^[[3]](#footnote-3)^ | Quality of studies included^[[4]](#footnote-4)^ | Assessed alignment with standards for ethical research ^[[5]](#footnote-5)^ |
| --- | --- | --- | --- | --- | --- | --- | --- | --- | --- | --- |
|  | [Van Schaik KD and Thompson SC [76]](#_ENREF_76)  *Indigenous beliefs about biomedical and bush medicine treatment efficacy for Indigenous cancer patients: a review of the literature*  Harvard University, Medical School, USA | Review of the literature  P: NS  K=NR: qualitative, a retrospective cohort study | Psychosocial: Cancer | ATSI: NS | Provide recommendations to healthcare providers about accommodating Indigenous beliefs when treating cancer | Identified five themes of Indigenous beliefs about treatment efficacy for cancer. Stated guidelines for healthcare providers to promote culturally safe and effective care for Indigenous patients. Call for more research integrating Indigenous healing beliefs and biomedical approaches to cancer treatment | No | No | No | No |
|  | [McDonald E, Bailie R, Brewster D and Morris P [19]](#_ENREF_19)  *Are hygiene and public health interventions likely to improve outcomes for Australian Indigenous children living in remote communities? A systematic review of the literature*  Charles Darwin University, Menzies School of Health Research, Australia | Systematic review  P:Up to 31 December 2003  K=19: clinical control trial (n=1), randomised control trials (n=9), controlled before and after (n=9) | Health behaviour: Hygiene and hand washing | ATSI Children: NS | Inform the development of hygiene programs by determining intervention/s reducing the incidence of skin, diarrhoeal and infectious diseases in Indigenous and developing countries populations | Established strong evidence of effect of education and handwashing with soap in preventing diarrhoeal disease. Overall, multifaceted interventions (which target handwashing with soap and include water, sanitation and hygiene promotion) are likely to provide the greatest opportunity to improve child health outcomes in remote Indigenous communities | No | Yes | Yes: Cochrane Collaboration Effective Practice and Organisation of Care Review Group (EPOC) guidelines. Generally poor | No |
|  | [Deek H, Abbott P, Moore L, Davison J, Cameron S, DiGiacomo M, McGrath SJ, Dharmendra T and Davidson PM [59]](#_ENREF_59)  *Pneumococcus in Indigenous peoples: The role of Indigenous health workers and implications for nursing practice*  American University of Beirut, Lebanon | Integrative literature review  2000-2013  K=33: qualitative | Biological and Healthcare system: Respiratory system | ATSI: NS | Describe the importance of immunizing against pneumonia and suggest strategies for screening and follow-up | Established a critical role of Indigenous Health Workers /nurses in improving the health outcomes and reducing health disparities | No | No | No | No |
|  | [Day A and Francisco A [20]](#_ENREF_20)  *Social and emotional wellbeing in Indigenous Australians: identifying promising interventions*  Deakin University, School of Psychology, Australia | Systematic review  From 1992 onwards  K=unclear: qualitative, quantitative, randomised trials, interventions | Psychosocial: Social and emotional wellbeing | ATSI: NS | Bring together knowledge in establishing the outcomes of programs that have the potential to improve the wellbeing | Small number of relevant program evaluations made impossible to articulate efficacy. Need for developing key indicators of improvement in SEWB, such that more robust evidence about program outcomes can be gathered. Further need to develop a broader framework to approach low levels of SEWB: the concepts of ‘grief and loss’, ‘healing’ and how high levels of social disadvantage impact on service utilisation and outcomes | No | No | Yes: Maryland scale rating according to the strength of internal validity. Average score of 16 programs 1.6 (max score 5) | No |
|  | [Clelland N, Gould T and Parker E [21]](#_ENREF_21)  *Searching for evidence: what works in Indigenous mental health promotion?*  Menzies School of Health Research, Australia | Systematic review  January 1990 and October 2003 K=22: interventions | Psychosocial: Mental health | ATSI: NS | Identify attributes of effective mental health promotion interventions to assist with planning more effective mental health promotion interventions and dissemination of findings | Efficacy of Indigenous mental health promotion interventions was not determined due to lack of well-conducted intervention evaluations; weak designs and insufficient information. The initiatives focused on individual behaviour change, rather than the broader social, ecological or policy determinants. The interventions involved extensive Indigenous participation, thus supporting cultural relevance and community involvement | Yes | No | Yes/ An abbreviated version of Rychetnik and Frommer's framework. In general weak designs | No |
|  | [Heffernan E, Andersen K and Kinner S [45]](#_ENREF_45)  *The insidious problem inside: mental health problems of Indigenous People in custody*  Queensland Health, Australia | Systematic review of the quantitative literature  From 1980 onwards  K=8: quantitative | Psychosocial: Mental health | ATSI: NS | Collect evidence regarding the mental health status of Indigenous Australians in custody due to lack of the data | Mental health problems and rates of mental illness are extremely high. Reported the shortfall in both the quantity and quality of research regarding the mental health of Indigenous people in custody. Need for systematic research about nature and extent of mental health problems of Indigenous people in custody with a focus on social and emotional wellbeing and use a culturally informed methods such as [Schlesinger CM, Ober C, McCarthy MM, Watson JD and Seinen A [98]](#_ENREF_98) | No | No | No | No |
|  | [Arnold M, Moore SP, Hassler S, Ellison-Loschmann L, Forman D and Bray F [22]](#_ENREF_22)  *The burden of stomach cancer in Indigenous populations: a systematic review and global assessment*  Erasmus University Medical Center, Netherlands | Systematic review  Up to May 2013  K=57: quantitative | Biological and Health inequality: Stomach cancer | ATSI, Maori, Indigenous people from the circumpolar region, native Americans, Alaska natives, Mapuche: gender, age NS | To identify populations at increased risk of developing and dying from the cancer and highlighting trends for closer attention by public health and cancer prevention specialists | Higher burden of stomach cancer in Indigenous populations globally; particularly evident among Inuit and in Maori; in stark contrast to the decreasing global trends. Need for close surveillance and further research of potential risk factors. Improving nutrition, housing sanitation, Helicobacter pylori eradication programmes could reduce stomach cancer rates; policies addressing these initiatives could reduce inequalities in stomach cancer burden | Yes | Yes | No | No |
|  | [Rumbold AR and Cunningham J [77]](#_ENREF_77)  *A Review of the Impact of Antenatal Care for Australian Indigenous Women and Attempts to Strengthen these Services*  Charles Darwin University, Menzies School of Health Research, Australia | Review  P: Up to February 2006  K=10: evaluations of interventions | Healthcare system: Childbirth | ATSI Women: NS | Assess impact on utilization and quality of care, birth outcomes and maternal views about care as a result of changes to services or changes in the delivery of antenatal care | The impact of the antenatal care programs evaluated and published to date remains inconclusive. Highlighted the need for good quality long-term data collection about the health services providing antenatal care for Australian Indigenous women | No | No | No | No |
|  | [Christian B and Blinkhorn AS [78]](#_ENREF_78)  *A review of dental caries in Australian Indigenous children: the health inequalities perspective*  University of Sydney, Australia | Literature review  P: NS  K=unclear | Health inequality: Dental care | ATSI Children: < 12, the main focus on the ages of 5-6 years for the primary dentition, and 12-year-olds for the permanent dentition | Provide information on prevalence, experience of dental caries, its disparity to support evidence-based program planning and policy-making | Reported high inequality as caries prevalence and experience particular in the primary dentition. The magnitude of the disparity was relatively unchanged over time. Highlighted the need for more research on the determinants of oral health inequalities and further funding | No | No | No | No |
|  | [Azzopardi PS, Kennedy EC, Patton GC, Power R, Roseby RD, Sawyer SM and Brown AD [23]](#_ENREF_23)  *The quality of health research for young Indigenous Australians: systematic review*  University of Melbourne, Murdoch Children’s Research Institute, Australia | Systematic review  P:1 January 1994 – 1 January 2011  K=360: descriptive (306 [85.0%]) included 14 case series, 37 qualitative, 93 surveillance studies, 149 cross-sectional, 4 case–control and 9 cohort studies; 54 evaluation studies including 34 (63.0%) quantitative | Indigenous health research | ATSI Children: aged 10–24 years | Assess the extent and quality of the evidence base related to the health and wellbeing of young Indigenous Australians in order to establish the health status and identify opportunities to improve their health outcomes | Data quality improved significantly over time but there are still some important gaps. Call for greater research investment in urban settings and with regard to mental disorders and injury, with a further emphasis on trials of preventive and clinical intervention. The findings provide a framework to allow Indigenous communities, researchers, funding bodies and the NHMRC to consider priorities for future research | Yes | Yes | Yes/ Not specified/ qualitative description, no scores provided. 69.4% of studies were graded as good quality | No |
|  | [Brooke NJ [24]](#_ENREF_24)  *Needs of Indigenous clients residing in Australian residential aged-care facilities*  University of Tasmania, Australia | Systematic review  P: 1995 to May 2010 inclusive  K=34: interventions | Healthcare system: Residential aged-care facilities | ATSI: NS | Identify evidence-based practice guidelines to support the care needs of Indigenous clients residing in residential aged-care facilities | Provided guidelines to the care incl. collaborative and individual approach, cultural safety, Indigenous persons in care should not be considered ‘routine’, need to maintain a high level of commitment to consultation with communities. Developed a series of service delivery themes covering areas such as communication, care, palliative care, death, activities and environmental considerations | No | No | No | No |
|  | [McCalman J, Tsey K, Wenitong M, Wilson A, McEwan A, James YC and Whiteside M [56]](#_ENREF_56)  *Indigenous men's support groups and social and emotional wellbeing: a meta-synthesis of the evidence*  James Cook University, School of Indigenous Australian Studies, Australia | Meta-synthesis of the evidence  P: 2000-2008  K=6: qualitative: peer-reviewed papers (n=3) ; unpublished reports (n=2), book chapter (n=1) | Psychosocial: social and emotional wellbeing | ATSI Men, age group was not specified | Unclear | Men’s support groups may be saving costs through reduced expenditure on health care, welfare, and criminal justice costs, and higher earnings. Call for future research to demonstrate this empirically | No | Yes | No | No |
|  | [Clifford A, Pulver LJ, Richmond R, Shakeshaft A and Ivers R [79]](#_ENREF_79)  *Disseminating best-evidence health-care to Indigenous health-care settings and programs in Australia: identifying the gaps*  University of New South Wales, National Drug and Alcohol Research Centre, Australia | Review  P: January 1990 and August (week 1) 2007  K=11: interventions or evaluations | Healthcare system: best practice dissemination | ATSI: NS | Identify practical opportunities for improving the quality of preventive healthcare in Indigenous health-care settings and programs | 1. Effective partnerships between government and research agencies, health-care providers and Indigenous health-care services.2.Evaluating strategies to improve the uptake of secondary preventive interventions.3. Measures of healthcare delivery in Indigenous-specific health-care settings quantify the effect of dissemination strategies. Call for more research | No | No | No | No |
|  | [Harlow AF, Bohanna I and Clough A [25]](#_ENREF_25)  *A Systematic Review of Evaluated Suicide Prevention Programs Targeting Indigenous Youth*  James Cook University, School of Public Health, Australia | Systematic review  P: in September 2012  K=11: interventions or evaluations | Health behaviour: Suicide prevention | Indigenous Youth from Australia, Canada, New Zealand, US: NS | Identify predictors of program success as well as gaps in the research | Two promising approaches were identified: the use of community-integrated development and strong youth involvement. Need for significant improvements in study design and evaluation | No | No | Yes by study design, however the evaluation process is vaguely describes without specifying the tool, no scores provided | No |
|  | [Davidson PM, Jiwa M, DiGiacomo ML, McGrath SJ, Newton PJ, Durey AJ, Bessarab DC and Thompson SC [60]](#_ENREF_60)  *The experience of lung cancer in Indigenous peoples and what it means for policy, service planning and delivery*  University of Technology Sydney, Centre for Cardiovascular and Chronic Care, Australia | Integrative literature review  P: January 1995 to July 2010  K=37: unclear | Healthcare system: Lung cancer care | ATSI: NS | Inform future policy, practice and research by identifying reasons for poorer outcomes and lower levels of treatment compared with non-Indigenous peoples, and opportunities for early intervention | Highlighted factors: exposure to risk factors, cultural and spiritual values, remoteness and geographic characteristics, entrenched socioeconomic inequalities and racism contribute to reduced service access and poor outcomes. Need for multifaceted interventions, supported by enabling policies that target individuals, communities and health professionals | Yes | No | No | No |
|  | [Gould GS, Munn J, Watters T, McEwen A and Clough AR [57]](#_ENREF_57)  *Knowledge and Views About Maternal Tobacco Smoking and Barriers for Cessation in Indigenous and Torres Strait Islanders: A Systematic Review and Meta-ethnography*  James Cook University, School of Public Health, Tropical Medicine and Rehabilitation Sciences, Australia | Systematic Review and Meta-ethnography  P: through to March 2011  K=7: descriptive (n=6), conceptual study (n=1) | Health behaviour: Tobacco smoking | ATSI Women, age group was not specified | Identify key knowledge, attitudes, beliefs, and barriers around maternal smoking and cessation. Provide recommendations for targeted interventions | Social norms and stressors within the Indigenous community perpetuating tobacco use; insufficient knowledge of smoking harms; inadequate saliency of antismoking messages; and lack of awareness and use of pharmacotherapy. Indigenous Health Workers have a challenging role, not yet fulfilling its potential. Pregnancy is an opportunity to encourage positive change where a sense of a “protector role” is expressed. Offered recommendations for practice and policy to overcome the barriers including focusing on the pregnant smoker in context with her environment and social networks | No | Yes | Yes/ The Hawker appraisal tools/ Av. score – 289 (max 360). Av. for descriptive studies 283; av. for conceptual study 320 | No |
|  | [Power J, Grealy C and Rintoul D [26]](#_ENREF_26)  *Tobacco interventions for Indigenous Australians: a review of current evidence*  Urbis Pty Ltd, Social Planning and Social Research Unit, Melbourne, Australia | Systematic review  P: 2001-2007  K=12: interventions and evaluations (n=11), descriptive (n=1) | Health behaviour: Tobacco interventions | ATSI: NS | Identified gaps in recent evidence regarding smoking cessation interventions and update a previous study findings | Five more studies were included. Smoking cessation strategies targeted at the individual level such as nicotine replacement therapy and/or counselling are likely to be effective for Indigenous individuals who are motivated to quit. However, there is no evidence regarding interventions likely to be effective in encouraging more Indigenous Australians to access these quit support strategies. Call for further research | No | No | Yes by study designs/National Health and Medical Research Council (NHMRC) levels of evidence scale. The majority of studies were type IV (the lowest level of evidence) | Yes/ Applicability to Indigenous Australians rating scale developed by Ivers (2001). The highest level was A and being assigned to 2 out of 24 studies |
|  | [Ivers RG [27]](#_ENREF_27)  *A review of tobacco interventions for Indigenous Australians*  Menzies School of Health Research, Australia | Systematic review  P: from 1980 to March 2001  K=4: interventions | Health behaviour: Tobacco interventions | ATSI: NS | Discuss the likely effect of a range of tobacco interventions if conducted in the population of Indigenous Australians | There were a number of small tobacco programs that had been conducted, particularly in the area of health promotion (in particular, development of health promotion material). Training health professionals in delivering a brief intervention resulted in some changes to practice and the evaluation of the mainstream advertising campaign showed that following the campaign, knowledge about tobacco had increased. No programs had been run or evaluated specifically for Torres Strait Islander people. Call for more research and evaluation | No | No | Yes/National Health and Medical Research Council (NHMRC) levels of evidence scale. Two studies were type IV; two were not classified | Applicability to Indigenous Australians rating scale developed by Ivers (2001). Most of the studies were of good evidence level B |
|  | [Priest N, Mackean T, Waters E, Davis E and Riggs E [46]](#_ENREF_46)  *Indigenous child health research: a critical analysis of Australian studies*  University of Melbourne, McCaughey Centre, Australia | Systematic analysis of descriptive studies  P: in October 2005  K=217: cross sectional (n=179, 82.5%), cohort (n=21, 9.7%), qualitative (n=12, 5.5%) and mixed methods (n=2, 0.9%) designs | Psychosocial and Biological: child health research | ATSI Children: 0-18 years or their parents | Examine the three key issues of Indigenous involvement in research, domains of the life-course model and geographical location of studies for Indigenous child health and wellbeing research | Research predominantly addressed physical health with few studies addressing mental health and wellbeing or health determinants. Indigenous involvement in the research process was not apparent in 71.4% of studies, although it appears to be increasing. 67.2% of the studies were conducted in very remote areas. Highlighted issues to be addressed: better reporting of Indigenous involvement in research; exploration of social, emotional, cultural and spiritual dimensions of health and wellbeing; ensure that research is relevant to all contexts in which Indigenous children grow and develop. Call for more research regarding the needs of urban Indigenous children | No | No | No | No |
|  | [Morris PS [28]](#_ENREF_28)  *Randomised controlled trails addressing Australian Indigenous health needs: A systematic review of literature*  Menzies School of Health Research, Australia | Systematic review  P: to 1997 inclusive  K=13: randomised control trial (n=9), not randomised control trials (n=4) | Psychosocial and Biological: adult health research | ATSI: NS | Describe the frequency and design of controlled trails specifically addressing the health needs of Indigenous Australians | Psychosocial, Biological: child health research | No | No | No | No |
|  | [Nelson A, Abbott R and Macdonald D [80]](#_ENREF_80)  *Indigenous Austalians and physical activity: using a social–ecological model to review the literature*  University of Queensland, School of Human Movement Studies, Australia | Review  P: post-1990  K=unclear | Health behaviour: Physical activity | ATSI: NS | Challenge some of the ‘taken-for-granted’ ways of thinking about promoting or researching physical activity with Indigenous Australians | Socioecological models can be valuable tools for understanding and promoting issues related to physical activity engagement for a range of populations but they may require complementary critical insights, including those from Indigenous perspectives | No | No | No | No |
|  | [Guy RW, James S. Smith, Kirsty S. Su, Jiunn-Yih Huang, Rae-Lin Tangey, Annie Skov, Steven Rumbold, Alice Silver, Bronwyn Donovan, Basil Kaldor, John M. [29]](#_ENREF_29)  *The impact of sexually transmissible infection programs in remote Indigenous communities in Australia: a systematic review*  University of New South Wales, Kirby Institute, Australia | Systematic review  P: to April 2011  K=12: cross-sectional screening studies | Biological: Sexually transmissible infections | ATSI: NS | Inform a randomised trial to control sexually transmitted infections in remote Indigenous communities | In three of the four programs, there was some evidence that clinical best practice and well-coordinated sexual health programs can reduce STI prevalence in remote Indigenous communities | No | No | No | No |
|  | [Shah PS, Zao J, Al-Wassia H and Shah V [53]](#_ENREF_53)  *Pregnancy and Neonatal Outcomes of Indigenous Women: A Systematic Review and Meta-Analysis*  Mount Sinai Hospital, Department of Pediatrics, Canada | Systematic review and meta-analysis  P: inception to September 2009  K=38: cohort studies without control (n=7), unmatched comparative population (n=31) | Biological: Pregnancy and neonatal outcomes | Indigenous Women globally: age group was not specified | Report on associations and explore reasons for heterogeneities in results among various subgroup populations rather than infer causality | Indigenous women are at increased risk of adverse pregnancy outcomes especially preterm birth. Differences in outcomes exist based on the country of origin of Indigenous women, reasons for which should be investigated. Call for studies assessing confounders | No | Yes | Yes/ Checklist based on criteria for sample selection, exposure assessment, outcome assessment, confounder, analytical, and attrition biases (Shah & Zao, 2009). 89% of studies had an overall moderate risk of bias | No |
|  | [Gould GS, McEwen A, Watters T, Clough AR and van der Zwan R [58]](#_ENREF_58)  *Should anti-tobacco media messages be culturally targeted for Indigenous populations? A systematic review and narrative synthesis*  James Cook University, School of Public Health, Tropical Medicine and Rehabilitation Sciences, Australia | Systematic review and narrative synthesis  P: inception to October 2011  K=21: quantitative (n=9) incl. randomised control trial (n=4), database analysis (n=1), post-intervention survey (n=1), before and after studies (n=2); qualitative mix methods studies (n=12) | Health behaviour: Smoking | Indigenous people globally: NS | Identify any differences in effect according to whether the messages were addressed to the target population or aimed at the general population | Although Indigenous people had good recall of generic anti-tobacco messages, culturally targeted messages were preferred. Maori may be less responsive to holistic targeted campaigns. Culturally targeted internet or mobile phone messages appear to be as effective in American Indians and Maori as generic messages in the general population. There is little research comparing the effect of culturally targeted versus generic messages with similar message content in Indigenous people | Yes | No | Yes/ 1.Daly et al’s hierarchy of evidence (77% of qualitative studies were category 3 (top category 1); 2.Scottish Intercollegiate Guidelines Network checklists (67% of quantitative studies had a high risk of bias) | No |
|  | [Chang AB, Taylor B, Masters IB, Laifoo Y and Brown Alexander DH [47]](#_ENREF_47)  *Indigenous healthcare worker involvement for Indigenous adults and children with asthma*  Charles Darwin University, Menzies School of Health Research, Australia | Intervention review  P: intercept to January 2011  K=1: randomised control trial | Healthcare system and Biological: Asthma | ATSI Children and Adults with asthma (recurrent wheeze, dyspnoea or bronchodilator responsiveness) that responds to beta agonists | Determine whether involvement of an Indigenous healthcare worker (IHW) in comparison to absence of an IHW in asthma education programs, improves asthma related outcomes in Indigenous children and adults with asthma | The findings are consistent with data from several papers (Fisher 2009; Flores 2009;Krieger 2009; La Roche 2006) that have largely shown the beneficial effects of health or community workers involvement in minor ethnic groups with poorer asthma outcomes | No | Yes | Yes/Risk of bias assessment according to the Cochrane Handbook. The main risk of bias is in the lack of blinding in some outcomes and insufficient sample size for the primary outcome | No |
|  | [McCalman J, Tsey K, Bainbridge R, Rowley K, Percival N, O'Donoghue L, Brands J, Whiteside M and Judd J [49]](#_ENREF_49)  *The characteristics, implementation and effects of Indigenous health promotion tools: a systematic literature search*  James Cook University, Cairns Institute, Australia | Systematic literature search  P:2002-2012  K=74: descriptive (n=63), evaluations (n=11) | Health behaviour: health promotion tools | ATSI: NS | Determine: 1) what are the characteristics of tools; 2) how and where have tools been developed and implemented; and 3) what were the effects of tool implementation? | The small number and generally moderate quality of implementation and evaluation studies means that little is known about how tools work to strengthen Indigenous health promotion practice. Call for more research and long-term investment in research to review the current use of health promotion tools and the factors that are likely to enhance their implementation | No | Yes | Yes/ Effective Public Health Practice Project (EPHPP) tool – for quantitative studies; Critical Appraisal Skills Program (CASP) tool – for qualitative studies. The quality of evaluation studies was strong for 7% of the studies | No |
|  | [Clifford AC, Doran CM and Tsey K [30]](#_ENREF_30)  *A systematic review of suicide prevention interventions targeting Indigenous peoples in Australia, United States, Canada and New Zealand*  Institute for Urban Indigenous Health, Australia | Systematic review  P: 1981 – 2012 (inclusive)  K=9: intervention evaluations | Health behaviour: Suicide prevention | Indigenous peoples in Australia, United States, Canada and New Zealand: NS | Determine the extent to which suicide prevention interventions are effective for reducing rates of Indigenous suicide and suicidal behaviours | Identified intervention strategies incl. Community Prevention, Gatekeeper Training, and Education. 33% of the studies measured changes in rates of suicide or suicidal behaviour, all of which reported significant improvements. Methodological quality issues: weak study designs, reliance on self-report measures, highly variable consent and follow-up rates, and the absence of economic or cost analyses. Call for evaluations of preventive interventions in order to construct one coherent suicide prevention program | No | Yes | Yes/ Effective Public Health Practice Project Quality Assessment Tool for Quantitative Studies. None had consistently strong methodology across the majority of applied criteria | No |
|  | [McCalman J, Tsey K, Clifford A, Earles W, Shakeshaft A and Bainbridge R [50]](#_ENREF_50)  *Applying what works: a systematic search of the transfer and implementation of promising Indigenous Australian health services and programs*  James Cook University, Cairns Institute, Australia | Systematic search  P: 1992 – 2011  K=1311 | Healthcare system: Transfer and implementation health services and programs | ATSI: NS | Examine the extent to which studies considered processes of transfer and implementation within and across Indigenous communities and healthcare settings | Few studies (only 1.6%) focus on the process of transfer and implementation of Indigenous Australian health services or programs. Highlight a need for partnerships between researchers and health services to evaluate the transfer and implementation of Indigenous health services and programs using rigorous designs, and publish such efforts in peer-reviewed journals as a quality assurance mechanism | No | Yes | Yes/ Study design: peer-reviewed and/or experimental or non-experimental. None of the impact evaluations were based on experimental study designs | No |
|  | [McCalman J, Bridge F, Whiteside M, Bainbridge R, Tsey K and Jongen C [31]](#_ENREF_31)  *Responding to Indigenous Australian Sexual Assault: A Systematic Review of the Literature*  James Cook University, Cairns Institute, Australia | Systematic review  P:1993 - 2012  K=23: descriptions of programs (n=4), measurement (n=1), descriptive or reviews (n=18) | Healthcare system: Sexual assault | ATSI: NS | Inform the design and implementation of a new sexual assault counselling, referral, support, and/or medical services in the nearby Indigenous community, as well as tailoring of the existing regional service to increase accessibility to Indigenous clients | Currently insufficient evidence to confidently prescribe what works to effectively respond to Indigenous Australian sexual assault. Call for the research | No | Yes | Yes/ Study quality (intervention studies only). None intervention studies were identified | No |
|  | [Derrick GE, Hayen A, Chapman S, Haynes AS, Webster BM and Anderson I [16]](#_ENREF_16)  *A bibliometric analysis of research on Indigenous health in Australia, 1972-2008*  The University of Sydney, Sydney School of Public Health, Australia | Bibliometric analysis  P: 1 January 1972 to 31 December 2008  K=820 | Indigenous health research | ATSI: NS | It was hypothesised that Indigenous-related health research would grow at the same rate and have the same level of ‘citedness’ as other fields of health research | There has been positive growth in publications by 5.2% more compared to all fields of Australian research. However, Indigenous publications were cited significantly less. 25% of the publications were not exclusively related to Indigenous health, but as part of a wider population study | Yes | No | No | No |
|  | [Sanson-Fisher RW, Campbell EM, Perkins JJ, Blunden SV and Davis BB [2]](#_ENREF_2)  *Indigenous health research: a critical review of outputs over time*  University of Newcastle, Australia | Critical review  P: 1 January 1987 to 31 December 1988, 1 January 1997 to 31 December 1998, and 1 November 2001 to 30 November 2003  K= 1731 | Indigenous health research | Indigenous health in Australia, Canada, New Zealand and the United States: NS | To determine the number and nature of publications on Indigenous health in Australia, Canada, New Zealand and the United States) in 1987–1988, 1997–1998 and 2001–2003 | Research was predominantly descriptive (75%-92%) | No | No | No | No |
|  | [Eades SJ, Taylor B, Bailey S, Williamson AB, Craig JC and Redman S [90]](#_ENREF_90)  *The health of urban Indigenous people: insufficient data to close the gap*  Baker IDI Heart and Diabetes Institute, Australia | Brief review  P: 2004-2009  K=555: evaluative interventions (2%) | Indigenous health research | ATSI: NS | Increase our understanding of the extent to which recent original research publications in Australia address the health of urban Indigenous people and may contribute to guiding the policy and practice | Few studies (11%) have examined the health needs of Indigenous people who live in urban areas. The government has to pay due attention to the non-remote-living population. Call for more research is to test the impact of policies and programs. | No | No | No | No |
|  | [Morris PS [18]](#_ENREF_18)  *A systematic review of clinical research addressing the prevalence, aetiology, diagnosis, prognosis and therapy of otitis media in Australian Indigenous children*  Menzies School of Health Research, Australia | Systematic review  P:1970-97 inclusive  K=59: majority surveys | Biological: Otitis media | ATSI Children: NS | Identify and summarise all relevant evidence available from studies addressing the prevalence, aetiology, diagnosis, prognosis and therapy of otitis media | Exposure to other young children with chronic nasal discharge is likely to be an important factor. Call for research concerned with evaluation and generalisability of studies from different populations | No | No | No | No |
|  | [Dawson AP [81]](#_ENREF_81)  *Asthma in the Australian Indigenous population: a review of the evidence*  University of South Australia, Division of health science, Australia | Review of the evidence  P: database search in January 2003; the Internet search in March 2003  K=10: descriptive | Biological: Asthma | ATSI: NS | Determine the current evidence concerning asthma | Number of studies has been increasing over the past decade. There remain no consensus of scientific opinion around the prevalence and aetiology of asthma. Need for extensive consultation with Indigenous communities to determine priorities for asthma research. Call for well-funded high methodological quality and cultural appropriate design studies | No | No | Yes, Sackett et al hierarchy of evidence. All studies reside at low level of the hierarchy of evidence scale. Particularly lacking is quality studies on asthma management interventions. | Yes, the READER instrument (designed for use in general public) |
|  | [Procter NG [82]](#_ENREF_82)  *Parasuicide, self‐harm and suicide in Indigenous people in rural Australia: A review of the literature with implications for mental health nursing practice*  University of South Australia, School of Nursing and Midwifery, Australia | Review of the literature  P: unclear  K=unclear | Health behaviour: Parasuicide self-harm and suicide | ATSI: NS | Examine the literature on underlying mental health issues, as well as primary and secondary actions to try to minimize the risk related to this area | Mental illness and suicide should incorporate understanding of risk and the factors that are protective against them, while simultaneously developing, disseminating and implementing effective, culturally and linguistically appropriate interventions | No | No | No | No |
|  | [Calabria B, Clifford A, Shakeshaft AP and Doran CM [32]](#_ENREF_32)  *A systematic review of family-based interventions targeting alcohol misuse and their potential to reduce alcohol-related harm in Indigenous communities*  University of New South Wales, Australia | Systematic Review  P: 2003-2010 inclusive  K=19: intervention | Health behaviour: Alcohol Misuse | ATSI: NS | Identify which interventions appear most promising to reduce alcohol-related harm | Family-based approaches offer considerable promise for reducing alcohol-related harms among Indigenous peoples. Family-based interventions are more likely to be acceptable, appropriate, and effective for Indigenous people. Call for high-quality economic evaluations | No | Yes | Yes, Dictionary for the Effective Public Health Practice Project Quality Assessment Tool for Quantitative Studies. 84% of studies were rated as strong for data collection methods, selection bias was rated as weak for 47% of studies. Most studies did not control for confounders | No |
|  | [Bowen A, Duncan V, Peacock S, Bowen R, Schwartz L, Campbell D and Muhajarine N [51]](#_ENREF_51)  *Mood and anxiety problems in perinatal Indigenous women in Australia, New Zealand, Canada, and the United States: A critical review of the literature*  University of Saskatchewan, Canada | A critical review of the literature  P: January 1980 – March 2010  K=18: quantitative (n=16); qualitative (n=1); dissertation (n=1) | Psychosocial: Mood and anxiety | Perinatal Indigenous women in Australia, New Zealand, Canada, US: NS | Not clearly defined | Need for to identify the extent of the problem; adapt or develop culturally appropriate screening tools; increase understanding of culture and meaning in illness experience; identify outcomes of mental health problems; understand more about positive mental health in Indigenous women; ensure scientific rigour | No | No | No | No |
|  | [Fromene R, Guerin B and Krieg A [89]](#_ENREF_89)  *Australian Indigenous Clients with a Borderline Personality Disorder Diagnosis: A Contextual Review of the Literature*  Indigenous Wellbeing & Liaison Program, Northern mental health services, Australia | Contextual review of the literature  P: unclear  K=unclear | Psychosocial: Borderline Personality Disorder (BPD) | ATSI: NS | Provide more detailed and culturally appropriate context in which to view individuals | Suggested alternative possible interpretations of BPD symptoms for Indigenous Australians which need to be considered as part of any diagnosis and treatment. Call for more research | Yes | No | No | No |
|  | [Jervis-Bardy J, Sanchez L and Carney AS [48]](#_ENREF_48)  *Otitis media in Indigenous Australian children: review of epidemiology and risk factors*  Flinders University and Flinders Medical Centre, Australia | Review of epidemiology and risk factors  P: 1985-2012  K=unclear | Biological: Otitis media | ATSI Children: NS | Investigate the epidemiology of and risk factors for otitis media in Indigenous children | Despite awareness of the epidemiological burden of and risk factors for otitis media in Indigenous children, studies undertaken since 1985 demonstrate that otitis media remains a public health concern and a chronic disease which has significant and persistent effects on many individuals. Need for a major policy consideration | No | No | No | No |
|  | [Ospina MB, Voaklander DC, Stickland MK, King M, Senthilselvan A and Rowe BH [54]](#_ENREF_54)  *Prevalence of asthma and chronic obstructive*  *pulmonary disease in Indigenous and non-Indigenous populations: A systematic review and meta-analysis of epidemiological studies*  University of Alberta Hospital, Canada | Systematic review and meta-analysis  P: up to October 2011  K=8: cross-sectional analytical | Biological: Asthma and chronic obstructive pulmonary disease | Adult Native Americans, Canadian Indigenouss, Australian Indigenouss and New Zealand Maori vs non-Indigenous populations: NS | Evaluate differences in asthma and COPD prevalence between adult Indigenous and non-Indigenous populations | Indigenouss were more likely to report having asthma than non-Indigenouss (Prevalence (POR) 1.41), particularly Canadian Indigenouss (POR 1.80), Native Americans (POR 1.41) and Maori (POR 1.64). Australian Indigenouss were less likely to report asthma (prevalence 0.49). Call for epidemiological research evaluating the existence of respiratory health inequalities between Indigenous and non-Indigenous populations | Yes | Yes | Yes, Critical Appraisal of the Health Research Literature Prevalence or Incidence of a Health Problem by [Loney PL, Chambers LW, Bennett KJ, Roberts JG and Stratford PW [99]](#_ENREF_99). Overall moderate quality | No |
|  | [Minges KE, Zimmet P, Magliano DJ, Dunstan DW, Brown A and Shaw JE [33]](#_ENREF_33)  *Diabetes prevalence and determinants in Indigenous*  *Australian populations: A systematic review*  Baker IDI Heart and Diabetes Institute, Australia | Systematic review  P: January 1997 and January 2010  K=24 | Biological: Diabetes | ATSI: NS | Clarify overall patterns of the prevalence of diabetes and impaired glucose tolerance (IGT), by determinants such as age, gender, region, ethnicity and remoteness | Although the prevalence of diabetes (up to 33.1%) and IGT (up to 21.1%) is high, there appears to be considerable variation in prevalence between different segments of the Australian Indigenous population. Data on diabetes prevalence in the urban Australian Indigenous population is lacking | No | No | No | No |
|  | [Porter C, Skinner T and Ellis I [34]](#_ENREF_34)  *The current state of Indigenous and Indigenous women with diabetes in pregnancy: A systematic review*  University of Western Australia, Combined Universities Centre for Rural Health, Australia | Systematic review  P: in October 2011  K=42 | Biological: Diabetes in pregnancy (DIP) | Indigenous Women globally: age group was not specified | Determine the prevalence of DIP and its impact on maternal and newborn health outcomes | DIP prevalence is not the same for all Indigenous and Indigenous women, e.g. Australian Indigenous 8.4% compared with 2–5% worldwide. Inconsistent study design without robust data is interfering with accurate prevalence of DIP | No | No | No | No |
|  | [Woods JA, Katzenellenbogen JM, Davidson PM and Thompson SC [35]](#_ENREF_35)  *Heart failure among Indigenous Australians: a systematic review*  University of Western Australia, Combined Universities Centre for Rural Health, Australia | Systematic review  P: from 1990 to April 2010  K=21 | Biological and Healthcare system: Cardiovascular diseases | ATSI: NS | Provide evidence on epidemiology and adequacy of relevant health service provision in Indigenous Australians | Despite the evident disproportionate burden of heart failure (in Central Australian Indigenous adults (5.3%) the accuracy of estimation from administrative data is limited by poor Indigenous identification, inadequate case ascertainment and exclusion of younger subjects from mortality statistics. Data suggest that undiagnosed cases may be common in this population. Call for more research | No | No | No | No |
|  | [McNamara BJ, Gubhaju L, Chamberlain C, Stanley F and Eades SJ [36]](#_ENREF_36)  *Early life influences on cardio-metabolic disease risk in Indigenous populations—what is the evidence? A systematic review of longitudinal and case–control studies*  Baker IDI Heart and Diabetes Institute, Australia | Systematic review  P: intercept to 8 March 2012  K=50 | Biological: Cardio-metabolic disease | Indigenous populations from Australia, Canada, New Zealand and the USA: NS | Assess the published evidence for the developmental antecedents of disease risk among Indigenous populations across the lifespan | Interventions to reduce the burden should focus on improving maternal health. Call for research towards potential protective actions, such as breastfeeding | Yes | No | Yes/ Risk of bias assessment tool designed by Shah PS et al 72% a low risk of bias, 18% had a high risk of bias | NR |
|  | [Graham S, Guy RJ, Cowie B, Wand HC, Donovan B, Akre SP and Ward JS [55]](#_ENREF_55)  *Chronic hepatitis B prevalence among Indigenous Australians since universal vaccination: a systematic review and meta-analysis*  University of New South Wales, Kirby Institute, Australia | Systematic review and meta-analysis  P: up to March 2011  K=12 | Biological: Hepatitis B (HBsAg) | ATSI: NS | Estimate higher rates of chronic hepatitis B | The disparity of HBsAg prevalence between Indigenous and non-Indigenous people has decreased over time; particularly since the HBV vaccination program in 2000. However HBsAg prevalence remains four times higher among Indigenous compared with non-Indigenous people. Need for opportunistic hepatitis B virus screening to identify people who would benefit from vaccination or treatment | No | Yes | No | No |
|  | [Carey TA, Wakerman J, Humphreys JS, Buykx P and Lindeman M [37]](#_ENREF_37)  *What primary health care services should residents of rural and remote Australia be able to access? A systematic review of “core” primary health care services*  Flinders University and Charles Darwin University, Centre for Remote Health, Australia | Systematic review  P: unclear  K=19 | Healthcare system: Availability of services | ATSI: NS | Delineate primary health care core services that residents of rural and remote Australia be able to access | Due to the variability of results in the 19 papers reviewed, it was not possible to delineate a universal or definitive set of core services. Synthesised characteristics: a suite of core primary health care services should be fit-for-purpose, relevant to the context, and its development should be methodologically clear, appropriate, and evidence-based | No | No | Yes  “Is this study underpinned by a strong body of knowledge?” and “Does the method accord with the objectives of the study?”  No data found | No |
|  | [Chamberlain C, McNamara B, Williams ED, Yore D, Oldenburg B, Oats J and Eades S [38]](#_ENREF_38)  *Diabetes in pregnancy among Indigenous women in*  *Australia, Canada, New Zealand and the United*  *States: a systematic review of the evidence for screening in early pregnancy*  Monash University, School of Medicine, Nursing and Health Sciences, Australia | Systematic review  P: intercept to July 2012  K=145 | Biological: Diabetes in pregnancy (DIP) | Indigenous women in Australia, Canada, New Zealand and the United States: NS | Assess the level of evidence for early screening for gestational diabetes mellitus | There is sufficient evidence describing the epidemiology (n=145 studies) of diabetes in pregnancy but insufficient evidence to address understanding current screening practice and rates (n=7); acceptability of GDM screening (n=0); efficacy and cost of screening for GDM (n=3); availability of effective treatment after diagnosis (n=6); and effective systems for follow-up after pregnancy (n=5). Need for evidence | No | No | Yes/ Quality of evidence for each statement and study references (H, high; M, moderate; L, low; VL, very low). Quality and quantity of evidence for Indigenous women is significantly limited | No |
|  | [Wakerman J, Humphreys JS, Wells R, Kuipers P, Entwistle P and Jones J [39]](#_ENREF_39)  *Primary health care delivery models in rural and remote Australia – a systematic review* Flinders University and Charles Darwin University, Centre for Remote Health, Australia | Systematic review  P: 1993–2005  K=93 | Healthcare system: delivery models | ATSI: NS | Describe what health service models were reported to work, where they worked and why | Sustainable models are able to address diseconomies of scale which result from large distances and small dispersed populations. Highlight a number of exemplary models of PHC service delivery which have been evaluated and shown to be successful | No | No | No | No |
|  | [Adegbija OO and Wang ZQ [15]](#_ENREF_15)  *Gender variations in waist circumference levels between Indigenous and non-Indigenous Australian populations: A systematic review*  University of Queensland, Centre for Chronic Disease, Australia | Systematic review and meta-analysis  P: inception to June 2013  K=17: cross-sectional | Biological: Obesity | ATSI: NS | Test heterogeneity among Indigenous and non-Indigenous Australians | Although Indigenous women were shorter and had lower weight estimates, they had greater WC levels than Indigenous men. Need to understand and reduce the high prevalence of central obesity and related chronic diseases among Indigenous women | No | Yes | No | No |
|  | [Bonevski B, Randell M, Paul C, Chapman K, Twyman L, Bryant J, Brozek I and Hughes C [40]](#_ENREF_40)  *Reaching the hard-to-reach: a systematic review of strategies for improving health and medical research with socially disadvantaged groups*  University of Newcastle, School of Medicine & Public Health, Australia | Systematic review  P: up to May 2013  K= 116: Randomised controlled trial (n=9); non-RCT comparison of strategies or groups (n=14); descriptive study (n=48); qualitative study (n=16); case studies without data (n=28) | Indigenous health research | Socially disadvantaged groups: NS | Increase the amount of health research conducted with socially disadvantaged groups | Identified strategies: institutions need to acknowledge extended timeframes, plan for higher resourcing costs and operate via community partnerships | No | No | Yes/ Quality of the evidence base “Poor” for 79% | No |
|  | [Lyons JG, O'Dea K and Walker KZ [41]](#_ENREF_41)  *Evidence for low high-density lipoprotein cholesterol levels (HDL-C ) in Australian Indigenous peoples: a systematic review*  Baker IDI Heart and Diabetes Institute, Victoria Australia | Systematic review  P: 1950 – 2012  K=15: cross-sectional | Biological: Cardiovascular disease and diabetes | ATSI: NS | Establish whether low HDL-C in Australian Indigenous people findings could be generalized across populations and settings | Confirms very low mean HDL-C levels are common in Australian Indigenous populations living in rural and remote communities. Inverse associations between HDL-C and central obesity, diabetes prevalence and inflammatory markers suggest a particularly adverse CVD risk factor profile. Call for further investigation by gender | No | Yes | Yes/ Sanderson S, Tatt ID, Higgins JP tool. Most included studies were of high (41%) or moderate quality (59%)- none were of very high or of low quality | No |
|  | [Laws R, Campbell KJ, van der Pligt P, Russell G, Ball K, Lynch J, Crawford D, Taylor R, Askew D and Denney-Wilson E [42]](#_ENREF_42)  *The impact of interventions to prevent obesity or improve obesity related behaviours in children (0–5 years) from socioeconomically disadvantaged and/or Indigenous families: a systematic review*  Deakin University, Centre for Physical Activity and Nutrition Research, Australia | Systematic review  P: January1993– November 2013  K= 32 | Biological: Obesity | Children (0–5 years) from socioeconomically disadvantaged and/or Indigenous families | Improve obesity related behaviours | Future studies should focus on improving study quality, including follow-up of longer-term anthropometric outcomes, assessments of cost effectiveness, acceptability in target populations and potential for implementation in routine service delivery | No | Yes | Yes/ McMaster University quality assessment tool. Less than 10% of studies were high quality | No |
|  | [Miller A, Smith ML, Judd J and Speare R [43]](#_ENREF_43)  *Strongyloides stercoralis : Systematic Review of Barriers to Controlling Strongyloidiasis for Australian Indigenous Communities*  Griffith University, Indigenous Research Unit, Australia | Systematic review  P: up to 2012  K=16 | Biological: Strongyloides stercoralis infection | ATSI: NS | Contribute to the development of initiatives for prevention, early detection and effective treatment of strongyloidiasis | Identified five points of intervention: (1) develop reporting protocols between health care system and communities; (2) test all Indigenous Australian patients, immunocompromised patients and those exposed; (3) health professionals require detailed information on strongyloidiasis and potential for exposure; (4) to establish testing and treatment initiatives within communities; and (5) to measure and report prevalence rates specific to communities and to act with initiatives based on these results | Yes? | Yes | No | No |
|  | [Banbury A, Roots A and Nancarrow S [52]](#_ENREF_52)  *Rapid review of applications of e-health and remote monitoring for rural residents*  Southern Cross University, School of Health and Human Sciences, Australia | Rapid literature review  P: January 2002–June 2013  K=19 | Healthcare system: E-health | Rural residents | Identify the evidence relating to the impact of e-health on rural and remote communities and residents | E-health has the ability to increase access to services in rural and remote areas, substantially reduce travel costs and inconvenience to patients, and support professional development of health professionals. E-health should be implemented alongside change management processes | Yes | Yes | No | No |
|  | [Bainbridge R, Tsey K, McCalman J and Towle S [44]](#_ENREF_44)  *The quantity, quality and characteristics of Indigenous Australian mentoring literature: a systematic review*  James Cook University, Cairns Institute, QLD | Systematic review  P: January 1983 to October 2012  K=15: qualitative | Mentoring literature | ATSI: NS | Develop a general structure of Indigenous Australian mentoring that describes different forms of mentoring relationships and explains what works best | There was insufficient evidence to confidently prescribe a best practice model. Call for research explored the effectiveness of mentoring, captured its impact qualitatively or quantitatively, developed appropriate measures or assessed its cost-effectiveness | No | Yes | Yes/ CASP tool for qualitative study appraisal. Only one paper met both quality criteria of methodological quality | No |

Table 2: Summary table of evidence from the Search 2

| # | First author(s), year, title | Type of review^[[6]](#footnote-6)^, period searched (P), number of studies (K) and type of studies analysed | Health area | Target group: gender, age | Reasons for the review | Major findings (review authors’ conclusions) | Research impact | Review design^[[7]](#footnote-7)^ | Quality of studies included | Assessed alignment with standards for ethical research^[[8]](#footnote-8)^ |
| --- | --- | --- | --- | --- | --- | --- | --- | --- | --- | --- |
| 1 | [Dudgeon P, Walker R, Scrine C, Shepherd C, Calma T and Ring I [83]](#_ENREF_83)  *Effective strategies to strengthen the mental health and wellbeing of Indigenous people* | Literature review  P: 1992-November 2013  K=49 | Psychosocial: Mental health and wellbeing | ATSI:NS | Commissioned by the Closing the Gap Clearinghouse  Aim: review programs and initiatives that aim to address Indigenous mental health and social and emotional wellbeing | Established a need to investing in the sustainability, development, adaptation and reach of both preventative and early intervention programs and initiatives in Indigenous mental health and social and emotional wellbeing as a key contribution to the success of the Australian Government’s Closing the Gap agenda | No | No, but methodology was described | Yes/ adapted from NHMRC Evidence classification scale by McTurk et al. (2008) | Yes,  Appropriateness classification  The majority were ranked as strong |
| 2 | [Wise S [84]](#_ENREF_84)  *Improving the early life outcomes of Indigenous children: implementing early childhood development (ECD) at the local level* | Review  P: NS  K=NS | Psychosocial: Developmental outcomes in the early years of life | ATSI Children from before birth to 8 years of age, their parents and caregivers | Commissioned by the Closing the Gap Clearinghouse  Aim: review and synthesise knowledge relevant to localised ECD | Discussed the Localised ECD as a promising framework for service delivery. The cultural change required is considerable. | No | No | No | No |
| 3 | [Osborne K, Baum F and Brown L [85]](#_ENREF_85)  *What works? A review of actions addressing the social and economic determinants of Indigenous health* | Review  P: 2000-2013  K=NS | Psychosocial: Social and economic determinants of Indigenous health | ATSI:NS | Commissioned by the Closing the Gap Clearinghouse  Aim: review evidence relating to ‘what works’ to influence the social and economic determinants of Indigenous health, in order to reduce health inequities, and ultimately contribute to closing the life expectancy gap | Identified points as characteristics of success in programs and strategies to address key social and economic determinants of health for Indigenous Australians | No | No, but methodology was described | No | No |
| 4 | [Bowes J and Grace R [86]](#_ENREF_86)  *Review of early childhood parenting, education and health intervention programs for Indigenous children and families in Australia* | Review  P: NS  K=NS | Setting and contexts: Parenting, early childhood education | ATSI: early childhood is defined as the years from conception to school entry | Commissioned by the Closing the Gap Clearinghouse  Aim: provide a review of the intervention programs | Highlighted need for evidence-based programs and professional development of service providers | No | No, but methodology was described | Yes/ NHMRC Level of evidence scale. Most studies had level IV: evidence obtained from case series, either post-test or pretest/[and] post-test | No |
| 5 | [Ivers R [61]](#_ENREF_61)  *Anti-tobacco programs for Indigenous people* | Resource sheet  P: NS  K=NS | Health behaviour: Smoking | ATSI:NS | Commissioned by the Closing the Gap Clearinghouse  Aim: discuss the harms resulting from tobacco use , and evidence-based approaches to reducing this harm | Highlighted need for more program delivery and systematic support | No | No | No | No |
| 6 | [Sims M [87]](#_ENREF_87)  *Early childhood and education services for Indigenous children prior to starting school* | Review  P: NS  K=NS | Setting and contexts: Education | ATSI Children: prior to starting school | Commissioned by the Closing the Gap Clearinghouse  Aim: unclear | Established actioned to be done in Early Childhood Development | No | No | No | No |
| 7 | [Closing the Gap Clearinghouse [62]](#_ENREF_62)  *Healthy lifestyle programs for physical activity and nutrition* | Resource sheet  P: NS  K=NS | Health behaviour: Physical activity and nutrition | ATSI:NS | Commissioned by the Closing the Gap Clearinghouse  Aim: assess the evidence regarding the effectiveness of physical activity and nutrition programs | Intensive lifestyle programs have been shown to be effective. The settings in which lifestyle programs are delivered and who delivers them appear to contribute to their effectiveness. Sport can be used to promote healthy lifestyles. Small number of evaluations | No | No | No | No |
| 8 | [Closing the Gap Clearinghouse [63]](#_ENREF_63)  *Ear disease in Indigenous children* | Resource sheet  P: NS  K=NS | Biological: Ear disease | ATSI Children: NS | Commissioned by the Closing the Gap Clearinghouse  Aim: reviews past and current programs, research and strategies (both government and non-government) for the prevention and treatment of ear disease | Identified strategies to reduce incidence of ear disease. An effective approach to otitis media and hearing loss in young Indigenous children needs to be part of a comprehensive approach to family, maternal and child health and be embedded in coordinated primary care systems | No | No | No | No |
| 9 | [Closing the Gap Clearinghouse [64]](#_ENREF_64)  *Strategies and practices for promoting the social and emotional wellbeing of Indigenous people* | Resource sheet  P: NS  K=NS | Psychosocial: Social and emotional wellbeing | ATSI:NS | Commissioned by the Closing the Gap Clearinghouse  Aim: reviews and identify effective programs that aim to promote social and emotional wellbeing | Identified effective programs. Cultural affiliation and engagement, including being able to converse in their native language, were positively associated with Indigenous peoples’ social and emotional wellbeing | No | No | No | No |
| 10 | [Closing the Gap Clearinghouse [65]](#_ENREF_65)  *Strategies to minimise the incidence of suicide and suicidal behaviour* | Resource sheet  P: NS  K=NS | Health behaviour: Suicide prevention | ATSI:NS | Commissioned by the Closing the Gap Clearinghouse  Aim: review of policies and programs that aim to prevent suicide and suicidal behaviour | Identified a few effective programs. Non-Indigenous-specific programs from Australia and overseas need to be tested with Indigenous populations. Call for more evaluations of suicide prevention programs | No | No | No | No |
| 11 | [Ware V-A and Meredith V [66]](#_ENREF_66)  *Supporting healthy communities through sports and recreation programs* | Resource sheet  P: NS  K~30 | Health behaviour: Sports and recreation | ATSI:NS | Commissioned by the Closing the Gap Clearinghouse  Aim: review the available evidence of a range of sports and recreation programs in relation to their effects on supporting and building healthy communities | Outlined a range of successful practices as well as some broad principles for effectively implementing sports or recreation programs | No | No, but methodology was described | No | No |
| 12 | [Mildon R and Polimeni M [88]](#_ENREF_88)  *Parenting in the early years: effectiveness of parenting support programs for Indigenous families* | Review  P: NS  K=NS | Setting and contexts: Parenting in the early years | ATSI:NS | Commissioned by the Closing the Gap Clearinghouse  Aim: examine what is known about programs for Australian Indigenous families that effectively support parenting in the early years | Evidence is weak. The effectiveness dependents on features related to the intensity and duration of the program, content delivered, teaching strategies, target group, and worker characteristics, cultural characteristics. Call for further research | No | No | No | Yes |
| 13 | [Ware V-A [67]](#_ENREF_67)  *Housing strategies that improve Indigenous health outcomes* | Resource sheet  P: NS  K~60 | Setting and contexts: Housing | ATSI:NS | Commissioned by the Closing the Gap Clearinghouse  Aim: explore the mechanisms by which housing influences health outcomes, and a range of practices that can improve the condition | A large body of evidence demonstrates that programs that improve the condition of Indigenous housing are an effective and highly cost-efficient means of improving Indigenous health outcomes. Housing interventions must be designed and implemented in close consultation with the affected community | No | No | No | Yes |
| 14 | [Pholeros P and Phibbs P [68]](#_ENREF_68)  *Constructing and maintaining houses* | Resource sheet  P: NS  K=NS | Setting and contexts: Housing | ATSI:NS | Commissioned by the Closing the Gap Clearinghouse  Aim: the best approaches to the construction and maintenance of Indigenous housing | Need for a national database for new construction detailing individual dwellings | No | No | No | No |
| 15 | [Strobel N and Ward J [69]](#_ENREF_69)  *Education programs for Indigenous Australians about sexually transmitted infections and bloodborne viruses* | Resource sheet  P: NS  K=NS | Setting and contexts: Education | ATSI:NS | Commissioned by the Closing the Gap Clearinghouse  Aim: examine available evidence for the effectiveness of sexual health education programs | The best interventions are based on understanding of the behaviours, knowledge, beliefs and practices that they are trying to influence; a well-trained and resourced workforce to implement sexual health education programs in the community, clinical and school settings;, community interventions that are multifaceted, culturally appropriate and have guaranteed long-term funding are necessary | No | No | No | No |
| 16 | [Harrison L, Goldfeld S, Metcalfe E and Moore T [70]](#_ENREF_70)  *Early learning programs that promote children’s developmental and educational outcomes* | Resource sheet  P: NS  K=NS | Setting and contexts: Education | ATSI Children: NS | Commissioned by the Closing the Gap Clearinghouse  Aim: review international and Australian research evidence for the characteristics of early learning programs that are effective in promoting developmental and learning outcomes | International research evidence suggests that greater duration in high-quality early learning programs, taught by qualified, well-resourced and effective educators, is particularly beneficial. There have not yet been any rigorous trials of targeted early learning programs in Australia, particularly for Indigenous children | No | No | No | No |
| 17 | [Closing the Gap Clearinghouse [71]](#_ENREF_71)  *Increasing employment rates for Indigenous people with a disability* | Resource sheet  P: NS  K=NS | Setting and contexts: Employment with a disability | ATSI:NS | Commissioned by the Closing the Gap Clearinghouse  Aim: Assess and summarise evidence on the effectiveness of the programs | The most effective program to assist people with psychological disabilities (including the most severe forms of mental illness) to obtain and retain a job is IPS by a focus on commencing employment as soon as possible; close coordination of mental health treatment with employment assistance; and the availability of ongoing support to retain employment | No | No | No | No |
| 18 | [Ware V-A [72]](#_ENREF_72)  *Supporting healthy communities through arts programs* | Resource sheet  P: NS  K~30 | Setting and contexts: Education | ATSI:NS | Commissioned by the Closing the Gap Clearinghouse | Outlined a range of successful practices, as well as some broad principles for effectively implementing an arts program | No | No | No | No |
| 19 | [McCuaig L and Nelson A [73]](#_ENREF_73)  *Engaging Indigenous students through school-based health education* | Resource sheet  P: NS  K=NS | Setting and contexts: Education | ATSI Children: NS | Commissioned by the Closing the Gap Clearinghouse | School-based health education can be enhanced through clearer understandings of what works, for whom and in what circumstances | No | No | No | No |
| 20 | [Ware V-A [74]](#_ENREF_74)  *Improving the accessibility of health services in urban and regional settings for Indigenous people* | Resource sheet  P: NS  K~30 | Healthcare system: Services accessibility | ATSI:NS | Commissioned by the Closing the Gap Clearinghouse | Identified barrier: physical accessibility, affordability, appropriateness and cultural acceptability and discussed how they can be overcome | No | No | No | Yes |
| 21 | [Higgins D and Morley S [75]](#_ENREF_75)  *Engaging Indigenous parents in their children’s education* | Resource sheet  P: NS  K=NS | Setting and contexts: Education | ATSI:NS | Commissioned by the Closing the Gap Clearinghouse. Reasons unclear | Identified programs that have been demonstrated to improve Indigenous parents’ engagement incl. strategies: • including parents directly in programs • modelling the kinds of educational supports that parents can develop at home • addressing barriers to parental involvement (such as referring parents to services to address problems such as substance misuse; addressing social isolation; engaging parents in further education and training) • creating new family norms that prioritise the involvement of Indigenous parents in their children’s education. | No | No | No | No |

1. As authors specified [↑](#footnote-ref-1)
2. If Yes: name the tool or the assessment process [↑](#footnote-ref-2)
3. Yes if PRISMA or any other relevant guidelines for reporting systematic reviews or meta-analyses were named [↑](#footnote-ref-3)
4. If Yes: name the tool and reported quality [↑](#footnote-ref-4)
5. If Yes: name the tool and reported outcome [↑](#footnote-ref-5)
6. As authors’ specified [↑](#footnote-ref-6)
7. Yes if followed PRISMA or any other relevant guidelines for reporting systematic reviews or meta-analyses [↑](#footnote-ref-7)
8. If Yes: name the tool and reported outcome [↑](#footnote-ref-8)
